# Synergistic effect of seed roasting and green extraction on oxidative stability in black cumin oil^☆^

**DOI:** 10.1016/j.fochx.2026.104671

**Published:** 2026-10-22

**Authors:** Michael Zeugswetter, Tobias Pointner, Lisa Simettinger, Marc Pignitter

**Affiliations:** a Institute of Physiological Chemistry, Faculty of Chemistry, University of Vienna, 1090 Vienna, Austria; b Vienna Doctoral School in Chemistry (DoSChem), University of Vienna, Währinger Str. 42, 1090 Vienna, Austria

**Keywords:** Green extraction, Oxidative stability, Antioxidant capacity, Acid value, Shelf-life, Valorization

## Abstract

Cold-pressed black cumin oil (BCO) is prone to oxidative deterioration due to its high polyunsaturated fatty acid (PUFA) content and is known for elevated acid and peroxide values. Prior studies show seed roasting reduces acid values and increases oxidative induction time (OIT), yet the PUFA-rich BCO remains oxidation-susceptible. This study demonstrated that green extraction using unroasted seed press cake enriched carotenoids (+33%) and chlorophyll (+58%) in the oil, indicating transfer of lipophilic press-cake constituents. It also enhanced antioxidant capacity, and lowered primary oxidation products (peroxides −58%, conjugated dienes −10%) in BCO, though acid values rose likely due to enzymatic activity. Furthermore, seed roasting lowered acid values by 82% and boosted thymoquinone content, while combining roasting with green extraction significantly increased OIT compared to unoptimized roasted BCO. These novel findings highlight the synergistic potential of seed roasting in combination with green extraction to valorize press cake while addressing BCO stability challenges.

## Highlights

• Green extraction valorizes black cumin press cake for bioactive enrichment.
• Carotenoid content increased by 33%, chlorophyll by 58% in black cumin oil.
• Peroxide value was reduced by 58% and conjugated dienes by 10%.
• Roasting seeds lowered acid value by 82% and boosted thymoquinone content.
• Combined roasting + extraction synergistically extended oxidative induction time.

## Graphical abstract

**Figure:**
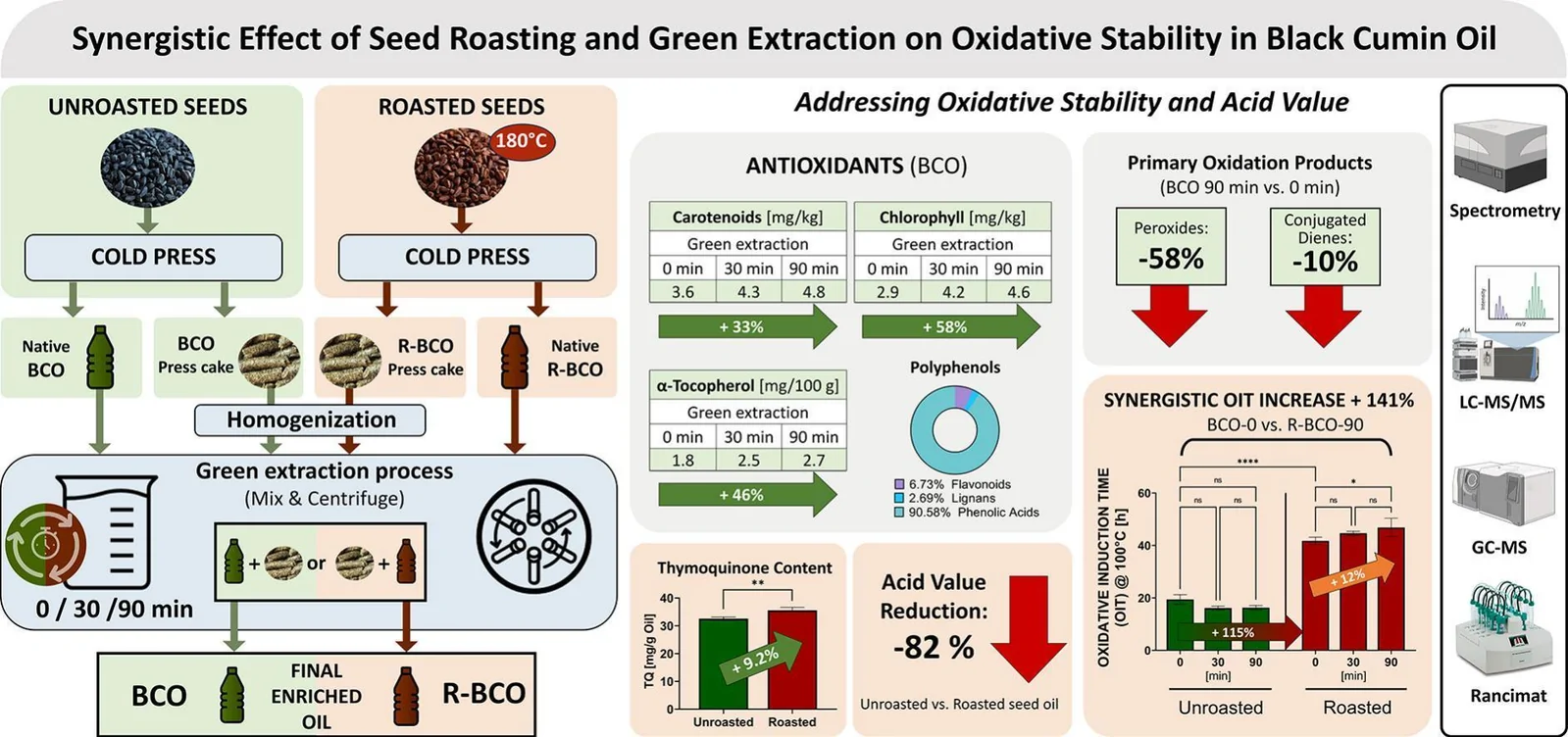

## 1 Introduction

Cold-pressed vegetable oils have attracted considerable attention among consumers due to their elevated concentrations of bioactive compounds and unsaturated fatty acids. Unlike conventional solvent extraction and refining processes, cold pressing is an energy-efficient and environmentally friendly method that retains higher levels of natural phenolic compounds, tocopherols, and carotenoids (Rathod et al., 2025). However, this high concentration of unsaturated fatty acids renders these vegetable oils highly susceptible to oxidative degradation (Gharby et al., 2025). While natural antioxidants inherent in the oil can counteract lipid oxidation, the overall oxidative stability of cold-pressed oils remains notably lower than that of refined oils (Kiralan et al., 2014). This reduced stability is primarily due to the presence of free fatty acids, which oxidize rapidly under the influence of temperature and light (Shahidi & Ying, 2010).

Oxidative stability is a critical indicator of oil quality, defined by the oil's resistance to oxidative degradation processes. Lipid oxidation occurs through several distinct chemical pathways, predominantly autoxidation, photooxidation, and enzymatic processes (Choe & Min, 2006). Autoxidation is a spontaneous free-radical chain reaction comprising initiation, propagation, and termination phases, wherein unsaturated fatty acids react with triplet oxygen to form unstable hydroperoxides. Alternatively, photooxidation is driven by light and photosensitizers, such as chlorophyll, which convert oxygen into a highly reactive singlet state that rapidly attacks double bonds (Pignitter et al., 2014). Furthermore, enzymatic oxidation is catalyzed by lipoxygenases that actively reduce iron atoms to form lipid radicals. Ultimately, these pathways cause primary oxidation products to break down into secondary compounds like aldehydes and ketones, which produce off-flavors and negatively impact consumer health (Hatcher et al., 2007).

To combat lipid oxidation, natural antioxidants play a pivotal role by protecting oxidizable lipids from degradation. Primary antioxidants, such as polyphenols and tocopherols, act as direct radical scavengers that donate hydrogen atoms to neutralize alkyl and peroxyl radicals, effectively interrupting the propagation chain reaction (Franchi et al., 1999). The efficacy of polyphenols in oils is frequently attributed to the polar paradox, which suggests that polar antioxidants exhibit high effectiveness in non-polar media like oil (Shahidi & Zhong, 2011). Secondary antioxidants function through indirect mechanisms such as metal chelation and oxygen quenching. For example, carotenoids and α-tocopherol are highly effective singlet oxygen quenchers. However, under certain conditions involving high oxygen availability, some oxidized antioxidants and chlorophylls can exhibit pro-oxidant behaviors, highlighting the complex chemical balance required to maintain oil stability (Choe & Min, 2006).

Black cumin (*Nigella sativa*) is a historically significant medicinal plant with origins in North Africa and Southern Europe. The seeds yield an oil highly concentrated in unsaturated fats, primarily linoleic acid (approximately 60%) and oleic acid (25%). The oil contains a unique array of volatile compounds, most notably thymoquinone (TQ). TQ is the primary bioactive constituent responsible for the oil's potent therapeutic, anti-inflammatory, and antimicrobial properties (Srinivasan, 2018). Beyond its fatty acid composition, black cumin oil (BCO) is exceptionally rich in polyphenols and tocopherols, contributing to its strong antioxidant capacity (Albakry et al., 2022). Nevertheless, the oil is highly susceptible to oxidative deterioration because it contains a large proportion of polyunsaturated fatty acids, especially linoleic acid, which are readily oxidized through autoxidation and photooxidation. In addition, endogenous lipases and lipoxygenases can increase free fatty acid formation and accelerate primary oxidation, leading to elevated acid and peroxide values as well as a significant decrease in both total polyphenolic content (TPC) and oxidation induction time (OIT) (Ashrafi et al., 2023; Tarasevičienė et al., 2023). These processes are further promoted by exposure to heat, light, and oxygen, while the naturally occurring pigments and bioactive compounds in the oil may act as either antioxidants or, under certain conditions, pro-oxidants. Therefore, improving the oxidative stability of BCO requires not only increasing protective bioactive compounds but also suppressing hydrolytic and oxidative degradation pathways.

Despite these valuable functional components, the oxidative stability of black cumin seed oil (BCSO) can still be severely compromised by environmental storage conditions.  A significant decrease in both total polyphenolic content (TPC) and oxidation induction time (OIT) was observed in BCSO stored in the dark at 4 °C for four months, while the acid value (AV) rose significantly during the same period (Tarasevičienė et al., 2023).

Enhancing the oxidative stability of cold-pressed oils without compromising their natural integrity remains a significant industrial challenge. Pretreatment of the seeds, such as controlled thermal roasting, can inactivate lipases and lipoxygenases, thereby improving oil yield and extending oxidative stability (Rękas et al., 2017). Various valorization strategies have recently been implemented to repurpose oil press cake, in particular “green extraction” methods are utilized to naturally enrich oils with antioxidants (Fruehwirth et al., 2020; Garcia-Mendoza et al., 2021; Pointner et al., 2025). Fruehwirth et al. (2020) demonstrated that the shelf life of cold-pressed linseed oil could be significantly increased by using the oil itself as an extraction medium to recover polyphenols directly from the press cake. After the oil pressing process is complete, a substantial amount of black cumin cake is generated as a nutrient-rich natural waste material containing abundant phenolics and bioactive compounds. In this paper, “green extraction” refers to the oil-assisted, solvent-free mixing of press cake with cold-pressed oil to transfer oil-soluble antioxidants into the oil.

Although seed roasting and green extraction each may improve BCO quality, their individual benefits are limited, which justifies combining both approaches. This study investigates whether black cumin press cake can be valorized through green extraction to enrich black cumin oil with naturally occurring bioactive compounds and improve oxidative stability. We hypothesized that the press cake still contains extractable antioxidants that can be transferred into the oil, thereby increasing antioxidant capacity and reducing primary oxidation products, although the acid value may remain unchanged. In parallel, we expected seed roasting to improve oil quality by lowering the acid value and increasing oxidative induction time without utilizing the antioxidant-rich press cake. We further tested whether combining roasting with green extraction yields a more stable oil than roasting alone. Overall, this work examines both the stabilization of black cumin oil and the reuse of press cake as a strategy to reduce waste and produce a more stable, bioactive-rich oil.

## 2 Materials and methods

### 2.1 Chemicals and materials

Chemicals and standards were obtained from Sigma-Aldrich Chemie Handels GmbH (Vienna, Austria): Folin–Ciocalteu's phenol reagent (2 N), gallic acid monohydrate (≥98.0%), anhydrous methanol (≥99.8%), tocopherols (α, γ, δ; all ≥96%), Trolox (97%), ABTS (≥98%), TPTZ (≥98%), potassium persulfate (≥99.0%), ammonium thiocyanate (≥97.5%), thymoquinone standard (≥98.5%), and caffeic acid, p-coumaric acid, ferulic acid, gallic acid, quercetin, and secoisolariciresinol (all ≥95%). Other suppliers were Merck KGaA (Darmstadt, Germany): n-hexane (≤95.0%) and FeSO4·7H2O (≥99.5%); Carl Roth GmbH & Co. KG (Karlsruhe, Germany): 2-propanol (≤99.95%, HPLC/LC-MS grade), diethyl ether (≥99.5%), ethanol (≥99.9%), phenolphthalein (1% in EtOH 90%), FeCl3 (≥98.5%), acetic acid (100%), and sodium acetate trihydrate (≥99%); VWR International GmbH: HPLC-grade methanol (≥99.9%), cyclohexane (≥99%), chloroform (≥99%), and isooctane (≥99.5%); Abcam: rac-tocol (>95%); Promega: DPPH; Mazola: sunflower oil.

### 2.2 Oil samples

Black cumin seeds (*Nigella sativa* L., cv. Znakharka), cold-pressed black cumin seed oil (BCO), and the corresponding press cake were supplied by Hagenthaler Ölmanufaktur (St. Andrä-Wördern, Austria). The seeds and oil originated from the same lot and were obtained from material cultivated in Lower Austria. According to producer specifications, the seeds had a moisture content of 8%, and their fatty acid composition comprised 15 g/100 g saturated fatty acids, 23 g/100 g monounsaturated fatty acids, and 62 g/100 g polyunsaturated fatty acids. All oil samples were kept at −80 °C before further processing.

### 2.3 Oil-assisted green extraction process

Following the protocol of Mazaheri et al. (2019a) black cumin seeds were roasted for 8 min at 180 °C by spreading them in a thin layer on a tray within a drying oven (DRY-line 56 prime, VWR International, Darmstadt, Germany). From these roasted seeds, additional cold-pressed oil samples (R-BCO) were subsequently produced using an electric oil press (OP 700 Emilio, Rommelsbacher, Dinkelsbühl, Germany). A press cake powder was generated by grinding the press cake from BCO and R-BCO samples for 2 min (Büchi mixer B-400, BÜCHI Labortechnik, Flawil, Switzerland). A mixture of press cake powder and oil was stirred continuously in a beaker (open system) using an Emerio HM-124178 250 W handheld mixer on highest speed (Emerio, B.V., Haarlem, The Netherlands) according to (Fruehwirth et al., 2020). Homogenization was performed for 30 min (BCO-30, R-BCO-30) and 90 min (BCO-90, R-BCO-90). The mixtures were subsequently centrifuged at 3200*g* for five min at 4 °C and the supernatant was filtered (Whatman qualitative filter paper, grade 1). Additionally, homogenization was performed with black cumin oil from unroasted seeds in closed 50 mL tubes (closed system). The procedure essentially followed the steps of the aforementioned procedure, with the exception that mechanical stirring *via* a handheld mixer was replaced by the continuous overhead rotation of an analog rotator RS-RR 5 (Phoenix Instrument, Garbsen, Germany) set at the highest speed for 30 min (C-BCO-30) and 90 min (C-BCO-90). To this end, the mixture comprising BCO (C-BCO-0) and finely pulverized press cake, was transferred into 50 mL centrifuge tubes. Although this mixing mode may differ in shear intensity from the handheld mixer used in the open system, it was selected to reduce oxygen exposure and potential volatile evaporation while maintaining continuous sample movement. For all extractions, 250 g press cake powder was uniformly mixed with 500 g oil. To prevent sensory and qualitative changes caused by exposure to light, temperature, and oxygen, the prepared sample material was hermetically sealed and stored in a freezer at −80 °C until analysis.

### 2.4 Antioxidants

#### 2.4.1 Determination of antioxidative capacity and total polyphenolic content

To determine the antioxidant capacity of the oil samples, two methods were applied: the Ferric Reducing Antioxidant Power (FRAP) assay and the 2,2′-Azino-bis(3-ethyl­benzothiazoline-6-sulfonic acid (ABTS) assay (Benzie & Strain, 1996; Re et al., 1999). Total polyphenolic content (TPC) was measured by Folin-Ciocalteu assay (Singleton et al., 1999). The preparation of the extracts was carried out identically for all tests according to Singleton et al. (1999). Briefly, 2.5 g oil sample were dissolved in 2.5 mL *n*-hexane and extracted three times with 1 mL of 80% methanol in water (*v*/v). Each step involved vortexing for 4 min followed by centrifugation (2280 *g*, 6 min, 4 °C) to separate the phases. The pooled lower aqueous phases (3 mL total) were then washed with 2.5 mL n-hexane, vortexed for 30 s, centrifuged, and the upper organic layer was discarded. The resulting methanolic extract was used for the subsequent assays.

ABTS and FRAP assays were performed as described by Re et al. (1999) and Benzie and Strain (1996). For the ABTS assay, ABTS solution (7 mM) and potassium persulfate (2.5 mM) were mixed and incubated overnight, then diluted with ethanol to an absorbance of 0.7 at 734 nm. Subsequently, 2 mL ABTS reagent were combined with 100 μL oil sample, and absorbance was measured in a cuvette at 734 nm using a Spark photometer (Tecan Group Ltd., Switzerland). Results were expressed as μmol Trolox equivalents (TE)/kg oil. For FRAP, the reagent contained acetate buffer (1.5 g sodium acetate trihydrate and 8 mL acetic acid in 500 mL), TPTZ solution (33 mg TPTZ in 10 mL of 40 mM HCl), and FeCl_3_ solution (20 mM), and measurements were recorded at 593 nm using the same instrument. Standards were prepared from FeSO_4_·7H_2_O. Total phenolic content (TPC) was determined in the phenolic extracts by adding 0.5 mL Folin-Ciocalteu reagent, 1 mL saturated sodium carbonate, and 6 mL water to the extracts, incubated for 90 min in the dark at room temperature, centrifuged (2280 *g*, 6 min, 4 °C), and the supernatant was measured at 765 nm. Results were expressed as mg gallic acid equivalents (GAE)/kg oil. Standard curves are shown in Figs. S1 and S2.

#### 2.4.2 Analysis of chlorophyll and carotenoids

For the analysis of carotenoids and chlorophyll according to Herchi et al. (2014), 1.5 g oil sample was mixed with 5 mL cyclohexane in a 15 mL tube. The mixture was homogenized by vortexing and centrifuged at 4 °C and 2280*g* for six min. The photometric measurement was performed in a cuvette, with the absorbance of chlorophyll measured at 670 nm and that of carotenoids at 470 nm. Pigment concentrations were calculated with the following formulas:

$$\text{Chlorophyll}\;\left[\mathrm{mg}/\mathrm{kg}\right]=\left(A_{670\mathrm{nm}}\times 10^{6}\right)/\left(613\times 100\times d\right)$$

$$\text{Carotenoid}\;\left[\mathrm{mg}/\mathrm{kg}\right]=\left(A_{470\mathrm{nm}}\times 10^{6}\right)/\left(2000\times 100\times d\right)$$

#### 2.4.3 Quantification of tocopherols by HPLC-UV

Oil samples were tested for tocopherol content using the method of Pignitter et al. (2014). Briefly, 50 mg oil were mixed with 1 mL 2-propanol in a 1.5 mL microcentrifuge tube (Eppendorf SE, Hamburg, Germany). Subsequently, 1 μL 5 mg/mL rac-tocol was added as an internal standard and the samples were filtered using a 0.2 μm PVDF filter. 10 μL were injected into the LC system (Shimadzu LC-20 CE, Shimadzu, Korneuburg, Austria) where water was used as solvent A and methanol as solvent B. Separation was achieved with a Shim-pack VP-ODS column (150 mm × 4.6 mm × 5 μm) and a diode array detector (Nexera X2 SPD-M30A) was used to analyze tocopherols at 295 nm. Quantification was carried out using α-, γ- and δ-tocopherol standards (Table S1).

#### 2.4.4 Quantification of polyphenols by LC-MS/MS

For the analysis of the polyphenol content using targeted Liquid Chromatography-Tandem Mass Spectrometry (LC-MS/MS), the method according to Pointner et al. (2024) was employed. For each sample, 2.5 g oil samples were weighed into 15 mL tubes and mixed with 3 mL of n-hexane and 1 mL of 80% methanol in water (*v*/v). After vortexing for four min, the samples were centrifuged (6 min, 2280*g*, 4 °C). The lower aqueous phase of each sample was collected and the extraction with methanol was repeated twice. The combined extract was washed with n-hexane, centrifuged (4 min, 2280*g*, 4 °C) and the hexane phase was discarded. The extract was then dried under nitrogen, dissolved in 500 μL methanol (80%) and mixed thoroughly. In the next step, the samples were filtered using a PVDF filter with a pore size of 0.2 μm and 10 μL of each sample were injected into an ESI-triple quadrupole LC-MS system (LCMS-8040, Shimadzu, Korneuburg, Austria). Separation was carried out *via* a Kinetex C18 column (Kinetex, 150 × 2.1 mm, 2.6 μm, Phenomenex, Aschaffenburg, Germany). Water with 0.1% formic acid served as mobile phase A, while phase B consisted of acetonitrile with 0.1% formic acid. The column temperature was kept constant at 35 °C, and the flow rate was 0.3 mL/min. The solvent B gradient began at 10%, increased linearly to 80% between 2 and 8 min, and reached 100% at 20 min. This 100% B concentration was held for 1 min, followed by a rapid decrease to 10% B, which was maintained for a 4-min re-equilibration period. Mass spectrometry was conducted *via* electrospray ionization (±4.5 kV) in positive and negative mode. Fragmentation utilized Ar collision gas (230 kPa) at an energy of ±30 V. N_2_ served as the nebulizing (3 L/min) and drying gas (10 L/min), with the desolvation line and heat block set to 150 °C and 350 °C, respectively. Multiple reaction monitoring (MRM, Table S5) data were processed using LabSolutions (v5.99 SP2, Shimadzu).

For data analysis, measured substances were divided into the polyphenol classes flavonoids (herbacetin), lignans (secoisolariciresinol) and phenolic acids (cinnamic acid, caffeic acid, *p-*coumaric acid, quinic acid). Quantification was carried out with the representative standards for the polyphenol classes: phenolic acids (caffeic acid, ferulic acid, gallic acid, *p-*coumaric acid), flavonoids (quercetin) and lignans (secoisolariciresinol) (Table S3). In the software Skyline v23.0 peak picking and alignment was performed to evaluate the resulting data as outlined in Pino et al. (2020).

### 2.5 Parameters of oxidative stability

#### 2.5.1 *Analysis* of conjugated dienes

Conjugated dienes (CD) were quantified following the method described by Pignitter et al. (2014). Briefly, 10 mg oil sample was dissolved in 10 mL of isooctane, and the absorbance was measured at 233 nm using a Spark spectrophotometer (Tecan Group Ltd., Switzerland). CD concentrations were calculated according to the Beer-Lambert law, employing a molar extinction coefficient (ε) of 25.250 M^−1^ cm^−1^ for linoleic acid hydroperoxide (Kūka et al., 2018).

#### 2.5.2 *Analysis* of non-enzymatic browning index

The non-enzymatic browning index (BI) for both unroasted and roasted BCO was assessed according to the methodology of (Suri et al., 2019). Oil samples were prepared by dilution in chloroform at a 1:20 (*w*/*v*) ratio. Then, absorbance measurements were taken at 420 nm using a Spark spectrophotometer (Tecan Group Ltd., Switzerland) and the absorbance values were used directly as the BI.

#### 2.5.3 Determination of acid value and free fatty acids

Acid value (AV) was determined following ISO 660:2020 (2020) with minor laboratory adaptations. Briefly, 3 g of oil were dissolved in 100 mL ethanol/diethyl ether (50,50, *v*/v) and heated at 40C for 45 s to ensure complete dissolution before titration. A 0.05 N KOH solution in 96% ethanol was used as titrant and phenolphthalein as an indicator. These modifications were introduced to improve sample handling and phase homogeneity for the highly viscous black cumin oil matrix, while the titration principle remained unchanged. The acid value was calculated from the volume of KOH consumed in mL (V), the concentration of the KOH solution in mol/L, the mass of the oil sample in g (m) and the molar mass of potassium hydroxide (56.1 g/mol) using Eq. 1.

(1) $$\mathrm{AV}=\frac{V\times c\times 56.1}{m}$$

Eq. 2 was used to calculate free fatty acids (FFA) in the samples:

(2) $$\mathrm{FFA}\left[\%\right]=\mathrm{AV}\times \frac{M_{\mathrm{oa}}}{10\times M_{\mathrm{KOH}}}$$

The formula includes the molecular mass of oleic acid (M_oa_ = 282.4 g/mol) as one of the dominant fatty acids in black cumin oil and the molecular mass of KOH (M_KOH_) (Dijkstra, 2016).

#### 2.5.4 Determination of peroxide value

The peroxide value (PV) was determined according to the International Dairy Federation (IDF) method, as described by Shantha and Decker (1994). Briefly, 25 mg oil was mixed with 9.9 mL chloroform/methanol (7,3, *v*/v) in a 15 mL centrifuge tube. Standards (0–40 μg Fe(III)/10 mL) were prepared by dilution of a 10 μg/mL Fe(III) chloride stock solution with the same solvent mixture (Fig. S3). The iron(II) chloride and ammonium thiocyanate reagents were prepared as described by Shantha and Decker (1994). For analysis, 50 μL iron(II) chloride solution and 50 μL ammonium thiocyanate solution were added sequentially to each sample or standard, mixed by vortexing, and incubated for 5 min before measurement of absorbance at 500 nm. PV was calculated using Eq. 3, which includes the absorbance of the sample (A_S_), the absorbance of the blank (A_B_), the slope of the standard curve (k), the molar mass of iron (55.84 g/mol), the sample weight in g (W) and a factor for converting oxygen to hydroperoxides (2).

(3) PVmeqO2kgoil=AS−AB×1k55.84×W×2

#### 2.5.5 Determination of oxidation induction time

Measurement of oxidation induction time (OIT) was performed according to the protocol of Aktar and Adal (2019). Each oil sample was weighed in quadruplicate (3 g each) into test tubes and exposed to an airflow of 20 l/h at 100 °C, 110 °C, and 120 °C using a Rancimat device (Metrohm 892, Herisau, Switzerland). The effect of temperature on lipid oxidation and the shelf-life of the oils was determined using the Arrhenius equation. Eq. 4 includes a frequency factor (A), the activation energy (E_a_ in kJ/mol), the gas constant (R in kJ × mol^−1^ × K^−1^), the temperature (T in K) and the experimentally determined rate constant of the reaction (k) (Aktar & Adal, 2019).

(4) $$\ln\left(k\right)=\ln\;A-\frac{E_{a}}{\mathrm{RT}}$$

By plotting ln(k) against 1/T (Arrhenius plot), the kinetic parameters E_a_  and A were determined from the slope and y-intercept of the linear regression (Table S8, Fig. S4 and S5). These parameters allowed for the extrapolation of the reaction rate constant at a storage temperature of 25 °C (k_25_). The predicted shelf life (SL) in months was subsequently calculated using Eq. 5, where the constants account for the conversion of hours into standard monthly units:

(5) $$\mathrm{SL}=\frac{1}{k_{25}\times 24\times 30.44}$$

#### 2.5.6 Determination of TQ and secondary oxidation products by SPME/GC–MS

TQ and secondary oxidation products in BCO were characterized *via* solid-phase microextraction coupled with gas chromatography–mass spectrometry (SPME-GC–MS), following the methodology of Holler et al. (2023). Briefly, 0.5 g of oil was weighed into 20 mL GC–MS vials. Volatiles were extracted in a GC-QP2010 Ultra system (Shimadzu, Korneuburg, Austria) using a divinylbenzene/ carboxen/polydimethylsiloxane fiber (DVB/C-WR/PDMS/10, Agilent Technologies, Santa Clara, USA) and separated on a Zebron ZB-5 ms column (30 m × 0.25 mm × 0.25 μm, Phenomenex, Torrance, USA). Samples were pre-equilibrated for 5 min at 50 °C prior to a 55-min headspace extraction. The fiber was subsequently desorbed in splitless mode within the injector port at 250 °C for 10 min, followed by a 5-min purge, while helium served as the carrier gas. Data acquisition was performed in full scan mode (*m*/*z* 40–350) using 70 eV electron impact ionization. The oven program started at 50 °C (5 min hold), increased at 4 °C/min to 300 °C, and concluded with a 30-min final hold. Identification was achieved by comparing recorded mass spectra against the NIST 17 Mass Spectral Library, using a match probability threshold of ≥85% (Holler et al., 2023). TQ was quantified using a standard curve prepared in sunflower oil, selected for its comparable fatty acid profile and lack of endogenous TQ (Table S2). Method validation parameters are shown in Tables S9 and S10. The secondary oxidation products, specifically hexanal and hexanol, underwent semi-quantification utilizing arbitrary units determined by calculating the peak area within the extracted ion chromatogram for the fragment at *m*/*z* 56.

### 2.6 Statistical analysis

Data normality and equality of variances were verified using the Shapiro-Wilk and Levene's tests respectively, prior to performing a one-way analysis of variance (ANOVA). Either Tukey's or Šídák's post-hoc test was used for multiple comparisons, as appropriate for the specific data set. For the analysis of the synergistic effect between roasting and green extraction, ordinary two-way ANOVA was applied with two levels of roasting status (unroasted and roasted) and three levels of extraction time (0, 30, and 90 min), together with the interaction term between both factors. Tukey's multiple comparisons test was then used to compare individual treatment groups. These data are presented in the supplementary material (Tables S11–S26). Conjugated dienes, ABTS and FRAP were measured only in oils obtained from unroasted seeds while browning index was also measured after roasting, but without green extraction. Therefore, these endpoints did not include the complete roasting × extraction-time factorial design and were not evaluated for the respective interaction. All experiments were conducted in triplicate (*n* = 3), except for rancimat measurements, where 4 replicates (*n* = 4) were analyzed. Results were expressed as the mean ± standard deviation (SD) and statistical significance was defined at *p* ≤ 0.05. Statistical processing and graphical representations were generated using GraphPad Prism (version 10.1.1).

## 3 Results and discussion

### 3.1 Antioxidant capacity and pigment-related changes

The antioxidant assays showed that green extraction affected the antioxidant capacity of BCO positively, indicating that the extraction process influenced different antioxidant-related endpoints (Fig. 1). In the ABTS assay, the oil exhibited a significant increase in antioxidant capacity, rising from 8225.13 ± 2.35 μmol TE/kg in BCO-0 to 8308.21 ± 2.35 μmol TE/kg after 30 min of green extraction (BCO-30), and further to 8991.80 ± 3.87 μmol TE/kg after 90 min (BCO-90). This pronounced increase suggests that green extraction enhanced the measurable radical-scavenging capacity of the oil when assessed by ABTS. The magnitude of these values is within the broad concentration range reported by Szydłowska-Czerniak et al. (2022), who measured 3800 μmol TE/kg to 14,000 μmol TE/kg in BCOs (Szydłowska-Czerniak et al., 2022).

**Fig. 1:**
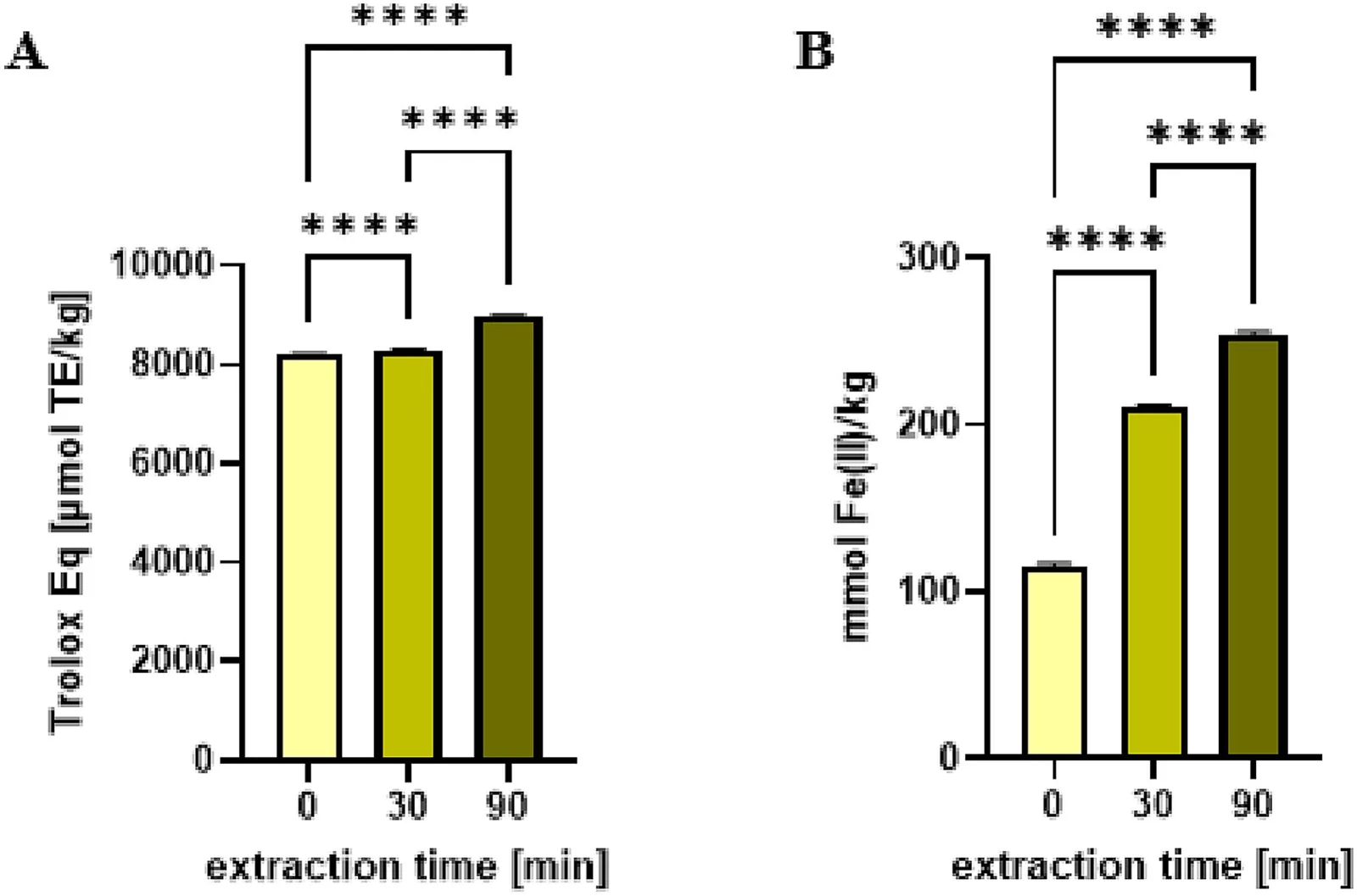
Impact of green extraction for 0, 30 and 90 min on the antioxidant capacity of black cumin oil from unroasted seeds, as determined by (A) ABTS and (B) FRAP assays. Data are expressed as mean ± standard deviation (*n* = 3). Statistical significance was assessed via one-way ANOVA followed by Tukey's post-hoc test: **** *p* < 0.001. (For interpretation of the references to colour in this figure legend, the reader is referred to the web version of this article.)

The high ABTS values may be explained by a high content of natural antioxidants in BCO, particularly polyphenols. This interpretation is consistent with the observations of Kostadinovic Velickovska and Mitrev (2013), who described a linear relationship between increasing phenolic compound content and increasing antioxidant capacity expressed as Trolox equivalents. In this context, the ABTS data fit well with the analytical results on the phenolic fraction of the oil, which also showed a comparatively high concentration of phenolic compounds in the samples (see chapter 3.2 and Fig. 5).

**Fig. 5:**
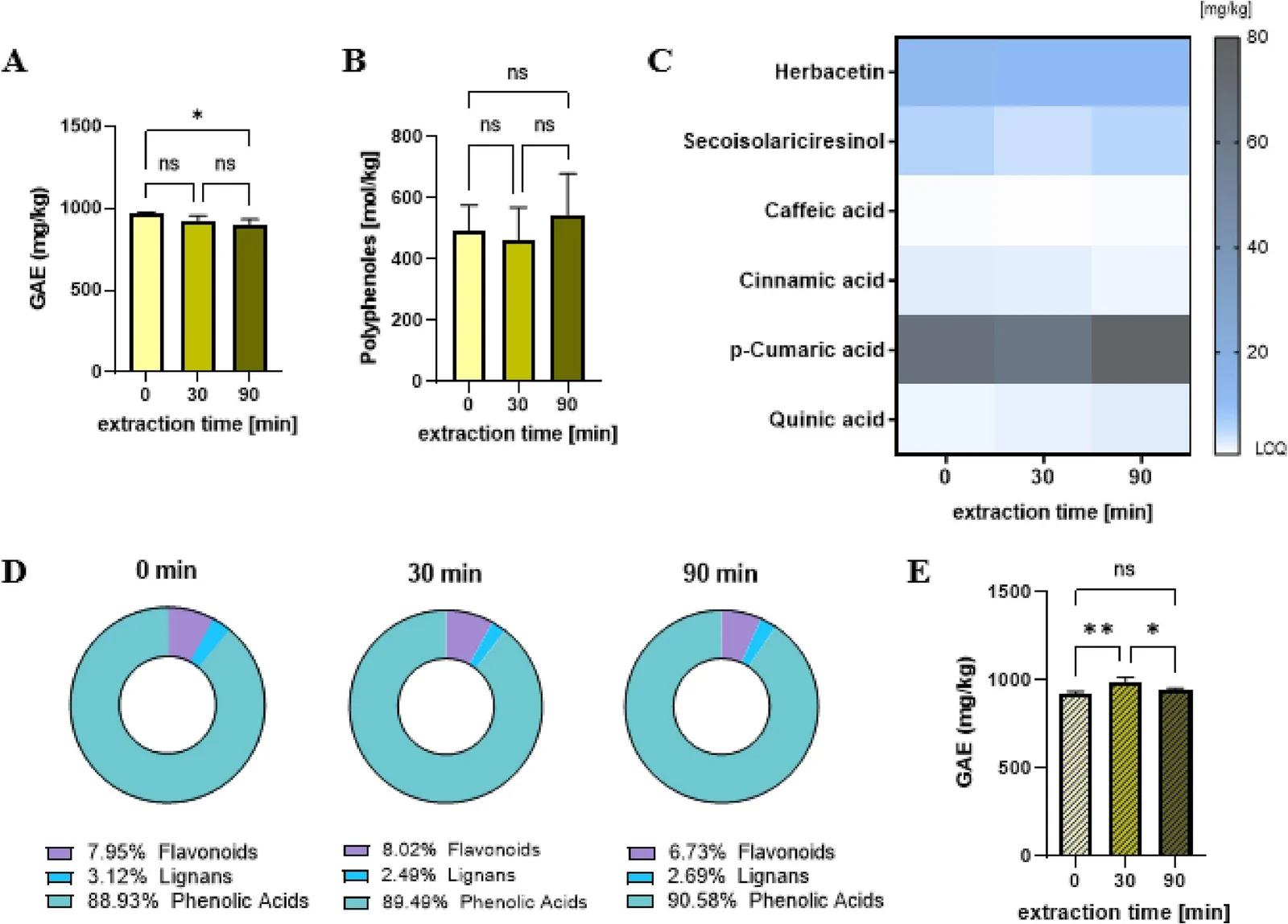
Total phenolic content (TPC) determined via (A) Folin-Ciocalteu assay and (B) LC-MS/MS analysis, alongside (C) individual phenolic profiles and (D) specific phenolic classes (flavonoids, lignans, and phenolic acids) in black cumin oil from unroasted seeds as well as Folin-Ciocalteu measurement of TPC in oil from roasted seeds (E) following green extraction for 0, 30, and 90 min. Data are expressed as mean ± standard deviation (*n* = 3). Statistical significance was assessed via one-way ANOVA followed by Tukey's post-hoc test: ns, non-significant; * *p* < 0.05. (For interpretation of the references to colour in this figure legend, the reader is referred to the web version of this article.)

The FRAP assay again showed a strong increase in reducing capacity after green extraction. The ferric reducing power increased from 115.32 ± 1.62 mmol Fe(II)/kg in BCO-0 to 210.84 ± 1.08 mmol Fe(II)/kg after 30 min (*p* < 0.0001) and to 253.99 ± 1.53 mmol Fe(II)/kg after 90 min (*p* < 0.0001). This highly significant increase indicates that green extraction enhanced compounds capable of reducing the ferric complex measured in the FRAP assay. This increase may be related not only to tocopherols, but also to higher concentrations of carotenoids. Müller et al. (2011) showed that some carotenes displayed approximately twice the antioxidant activity of α-tocopherol in the FRAP assay, while many xanthophylls showed ferric reducing capacities comparable to or above α-tocopherol (Müller et al., 2011). Furthermore, chlorophyll may also contribute substantially to the increase in FRAP values, because chlorophyll contains structural features such as phenolic rings and hydroxyl groups that can function as hydrogen donors (Oluwagunwa et al., 2024).

The pigment analysis results, presented in Fig. 2, support this interpretation and provide a mechanistic basis for the enhanced ABTS and FRAP responses observed following green extraction. The carotenoid concentration increased significantly from 3.65 ± 0.15 mg/kg oil in the initial sample to 4.34 ± 0.30 mg/kg after 30 min and 4.83 ± 0.40 mg/kg after 90 min. Although no significant difference was found between BCO-30 and BCO-90, the overall increase compared with the starting material demonstrates that green extraction promoted the transfer of carotenoid compounds into the oil. The measured concentrations are in the same general range as literature values, including 3 to 4 mg/kg oil in BCO of Iranian origin, while much lower values of 0.18 mg/kg oil were reported in Turkish BCO (Ashrafi et al., 2023; Kiralan et al., 2014).

**Fig. 2:**
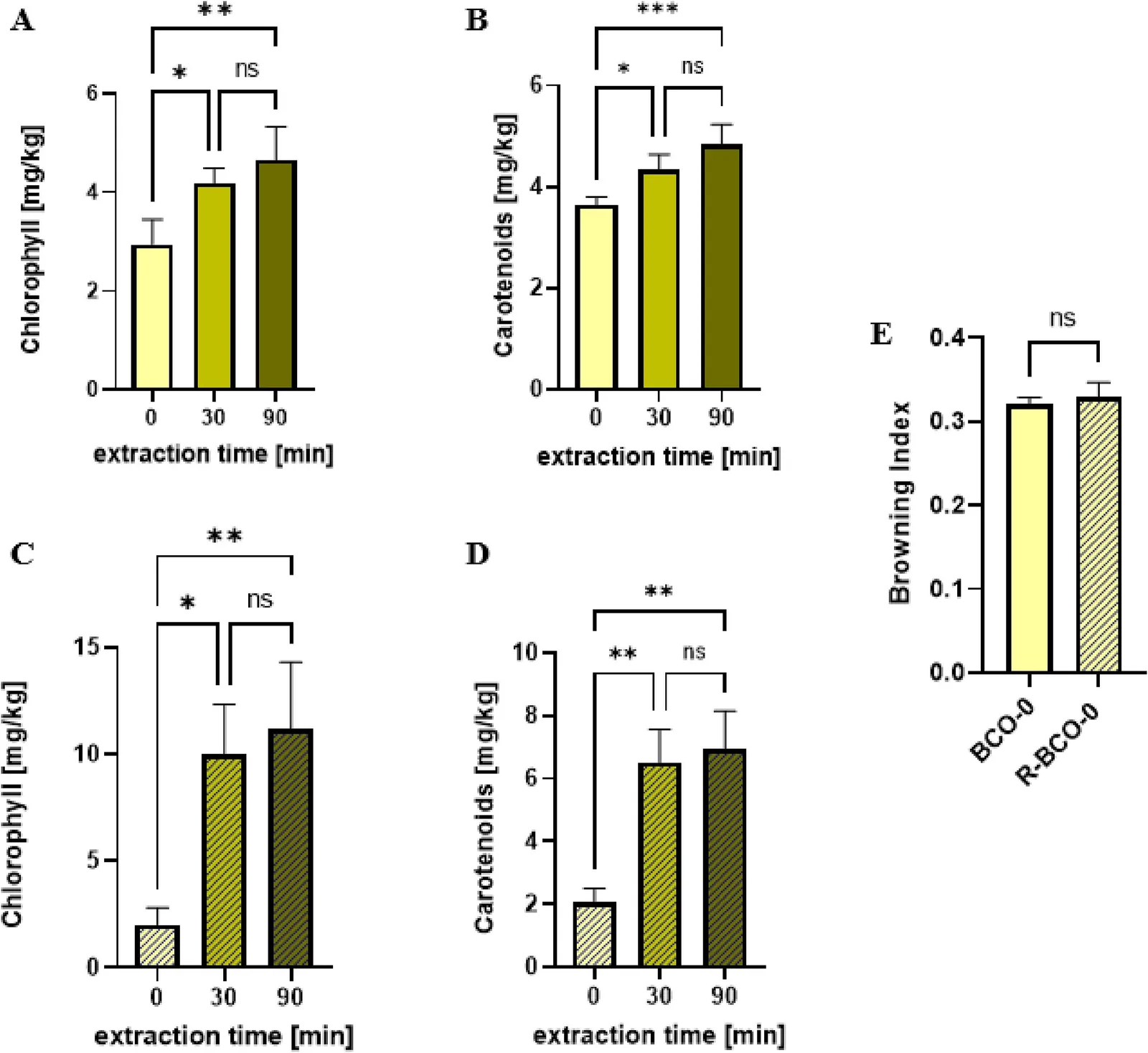
Analysis of pigments in oil samples. Contents of chlorophylls and carotenoids in black cumin oil from unroasted seeds (A, B) and roasted seeds (C, D) after green extraction for 0, 30 and 90 min, as well as browning index of native BCO and R-BCO (E) as determined by spectrophotometric analysis. Data are expressed as mean ± standard deviation (*n* = 3). Statistical significance was assessed via one-way ANOVA followed by Tukey's post-hoc test: ns, non-significant; * *p* < 0.05; ** *p* < 0.01; *** *p* < 0.001. (For interpretation of the references to colour in this figure legend, the reader is referred to the web version of this article.)

The chlorophyll content changed in a very similar way. The concentration increased from 2.95 ± 0.50 mg chlorophyll/kg in BCO-0 to 4.17 ± 0.32 mg/kg in BCO-30 and to 4.65 ± 0.68 mg/kg after 90 min of green extraction. As with carotenoids, no significant difference was detected between the two extraction times, but both extracted samples were clearly above the initial value. The chlorophyll concentrations measured in this work are comparable with literature values, including approximately 3 mg/kg and up to 6 mg/kg (Mazaheri et al., 2019a; Suri et al., 2019). There was a visually darker and more greenish appearance of the oil after green extraction, which can be explained by the higher carotenoid and chlorophyll concentrations, since carotenoids impart yellow to reddish tones whereas chlorophylls contribute brown-green coloration (Suri et al., 2019). Mechanistically, green extraction may enrich oil-soluble antioxidants by letting compounds retained in the press cake partition into the oil during prolonged mixing. Mechanical agitation and grinding increase contact area, shorten diffusion distances, and release antioxidants trapped in cell fragments, so lipophilic compounds move more easily into the oil. Cold pressing itself is too fast and leaves much of these compounds in the cake, which is why the later mixing step is more effective. Related work has shown that this approach can substantially increase chlorophyll, carotenoid, and tocopherol levels in flaxseed oil (Fruehwirth et al., 2020; Pointner et al., 2025).

In R-BCO, the carotenoid concentration increased from 2.05 ± 0.47 mg/kg in R-BCO-0 to 6.95 ± 1.19 mg/kg in R-BCO-90, while chlorophyll content rose even more markedly from 2.02 ± 0.80 mg/kg to 11.17 ± 2.39 mg/kg over the same extraction period. These increases were greater than those observed in BCO, indicating that roasting improved pigment extractability and thereby promoted a stronger transfer of these compounds into the oil during green extraction. This interpretation is consistent with previous reports showing that dry-air roasting under similar conditions can increase carotenoid and chlorophyll concentrations in cold-pressed black cumin oil, likely by enhancing pigment release from the seed matrix (Mazaheri et al., 2019a; Suri et al., 2019).

No significant difference in the browning index was observed between BCO-0 and R-BCO-0, suggesting that roasting under the applied conditions did not substantially increase Maillard-derived browning products in the oil. This is in contrast to the higher browning indices reported by Suri et al. after roasting at 180 °C, which may reflect differences in seed origin and growing conditions. Similar studies have also reported no significant increase in Maillard markers after roasting, including in vegetable oils examined for browning index and HMF formation (Ji et al., 2023). Overall, the pigment measurements in R-BCO indicate that roasting improved extractability of beneficial pigments without producing an excessive browning response.

### 3.2 Tocopherols and polyphenols

The HPLC analysis of tocopherols is shown in Fig. 3. Green extraction had only a limited effect on the total tocopherol concentration of BCO. The mean total tocopherol content in BCO-0 was 5.13 ± 0.30 mg/100 g, and although a slight increasing trend was visible with longer extraction time, no significant changes were detected. The reported value is comparable to values in the literature, for example 4.5 mg/100 g in BCO from Macedonia (Szydłowska-Czerniak et al., 2022).

**Fig. 3:**
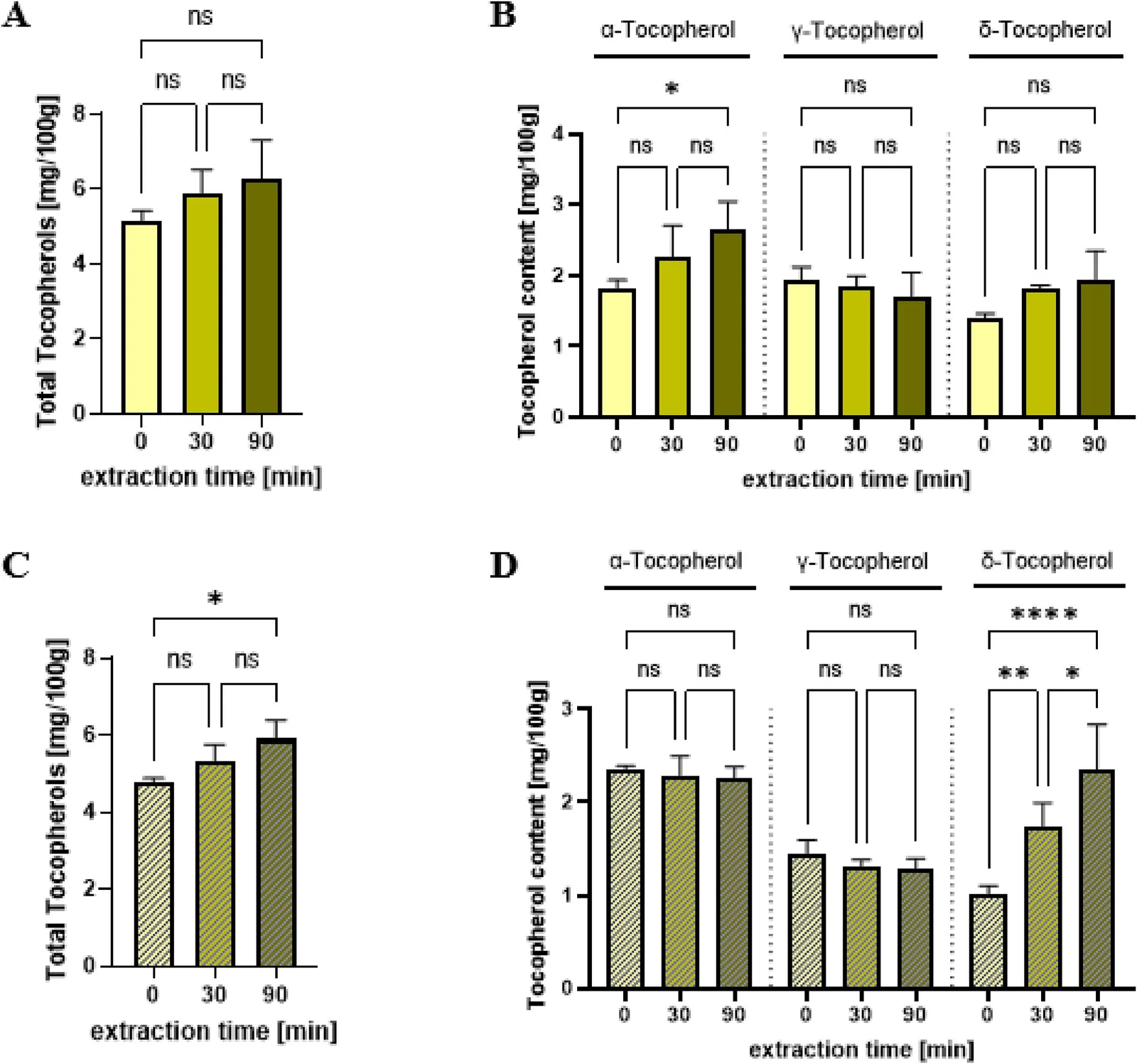
Total tocopherol content and individual tocopherol homologues (α-, γ-, and δ-tocopherol) in black cumin oil from unroasted seeds (A, B) and roasted seeds (C, D) following green extraction for 0, 30, and 90 min, as determined by HPLC-UV analysis. Data are expressed as mean ± standard deviation (*n* = 3). Statistical significance was assessed via one-way ANOVA followed by Tukey's post-hoc test for total tocopherols and with Šídák's post-hoc test for individual tocopherols: ns, non-significant; * *p* < 0.05; ** *p* < 0.01; **** *p* < 0.0001. (For interpretation of the references to colour in this figure legend, the reader is referred to the web version of this article.)

A more differentiated picture emerged when individual tocopherol fractions were considered. δ-tocopherol was present at 1.39 ± 0.07 mg/100 g in BCO-0, which is comparable to the low δ-tocopherol contents below 1 mg/100 g described by Matthaus and Özcan (2011) in 12 BCOs from Germany (Matthaus & Özcan, 2011). The γ-tocopherol content was 1.92 ± 0.19 mg/100 g in the initial state, and no significant effect of green extraction was detected for this fraction. The α-tocopherol concentration, however, responded differently: after 90 min of green extraction, it increased significantly from 1.81 ± 0.12 mg/100 g to 2.65 ± 0.39 mg/100 g. This result is important because it shows that green extraction did not affect all tocopherol homologues in the same way. This may indicate that α-tocopherol has been more readily mobilized from the press cake into the oil than γ- or δ-tocopherol. Furthermore, tocopherols may be associated with membranes or lipoproteins, which could make their transfer into BCO more difficult in general (Beldean-Galea et al., 2010; Fruehwirth et al., 2020). In R-BCO, no significant change was observed for α- or γ-tocopherol during green extraction, whereas δ-tocopherol increased in a time-dependent manner. This suggests that roasting altered the extractability of tocopherol homologues, potentially reducing the transfer of α-tocopherol while favoring δ-tocopherol enrichment in the roasted system. Consistent with this interpretation, the total tocopherol content increased significantly from 4.778 ± 0.106 mg/100 g in R-BCO-0 to 5.896 ± 0.486 mg/100 g in R-BCO-90. A significant decrease (*p* = 0.0455) was detected for α-tocopherol in this study comparing BCO-0 and R-BCO-0. Similar roasting-dependent shifts in tocopherol profiles have been reported for other oils, although the direction of change appears to be species dependent. Sun et al. (2022) observed a decrease in α- and δ-tocopherol in flaxseed oil after dry-air roasting, whereas Zhao et al. (2012) reported an increase in tocopherols in perilla seed oil after roasting.

The phenolic fraction of the oil was analyzed by two complementary approaches, and together these data point to a high but only modestly extractable phenolic pool (Fig. 5). Using the Folin-Ciocalteu assay, the TPC of BCO-0 was 963.90 ± 11.09 mg GAE/kg. After green extraction, a slight decline to 898.02 ± 35.69 mg GAE/kg was observed in BCO-90. This decrease may be attributable to oxidative and photochemical reactions that could lead to degradation of phenolic compounds (Singleton et al., 1999). The measured Folin-Ciocalteu values fall within the broad literature range of 315.68 ± 0.56 mg GAE/kg and up to 1400 mg GAE/kg (Albakry et al., 2022; Tarasevičienė et al., 2023). Additionally, storage can influence total phenolic levels, as Tarasevičienė et al. (2023) observed a decrease from 1401.9 mg GAE/kg to 1079.6 mg GAE/kg after four months of storage.

The Folin-Ciocalteu assay has limitations because it is not selective for phenolic compounds alone. The utilized reagent can also react with a broad range of reducing substances, not only phenolic hydroxyl groups. Relevant interferents include ascorbic acid, dehydroascorbic acid, reducing sugars such as glucose and fructose, and certain amino acids including tyrosine, whereas formic acid and acetic acid have also been reported to interfere in some matrices (Bastola 2017, Rangel 2013). Also, its reactivity varies substantially depending on the type of polyphenols present (Singleton et al., 1999). For this reason, the study complemented the photometric assay with an additional quantitative and qualitative LC-MS/MS analysis. The interfering compounds can reduce the Folin reagent and thereby bias TPC values upward or alter apparent trends during processing. In our system, the slight decrease observed by the Folin–Ciocalteu assay may therefore reflect changes in non-phenolic reducing constituents rather than a true loss of phenolic compounds. By contrast, the LC–MS/MS workflow used here is a targeted multiple-reaction-monitoring method that separates analytes chromatographically and detects specific ion transitions for individual phenolic standards, which minimizes cross-reactivity and allows direct quantification of individual polyphenols.

The LC-MS/MS data confirmed the presence of a high polyphenol concentration in BCO, but they also showed that green extraction did not significantly enrich the total polyphenol pool (Table S4). The mean polyphenol concentration in BCO-0 was 492.43 ± 85.31 mol/kg, and after 90 min of green extraction the mean increased only slightly to 543.02 ± 136.54 mol/kg, without statistical significance. The results suggest that the green extraction approach, which was effective for enriching polyphenols in linseed oil according to Fruehwirth et al. (2020) and Pointner et al. (2025), cannot be transferred to BCO to the same extent. A possible explanation is that BCO already contains a comparatively high TPC after cold pressing, so the remaining potential for further transfer of phenolics from the press cake into the oil may be limited. Because the LC–MS/MS data show a small, non-significant increase in summed polyphenol concentrations after green extraction while the Folin–Ciocalteu assay shows a slight decrease, we interpret these results as evidence that the actual phenolic content of BCO did not decrease during extraction. Instead, the Folin–Ciocalteu result is likely influenced by changing levels of other reducing constituents. Consequently, the main conclusions, that green extraction does not substantially change the total extractable phenolic pool of BCO but does transfer pigments and selected antioxidants and that roasting improves oil stability, remain supported by the more selective LC–MS/MS measurements.

The class distribution of phenolics gives additional insight into the composition of BCO (Fig. 5d). Phenolic acids accounted for about 90% of the TPC, making them the dominant phenolic class. This agrees with literature data, which also describe phenolic acids as the major phenolic constituents of BCO (Benazzouz-Smail et al., 2023). Lignans represented about 3% of the TPC in the oil, while flavonoids accounted for about 8%. Green extraction caused only minor and non-significant changes in these relative class proportions.

The qualitative LC-MS/MS profile further shows that the phenolic composition of the oil was dominated by *p-*coumaric acid (Fig. 5c). Its concentration increased slightly, though not significantly, from 67.29 ± 2.20 mg/kg in BCO-0 to 76.14 ± 9.87 mg/kg in BCO-90. This level can be classified as high, particularly compared to the low value of 0.09 ± 0.003 mg/kg reported by Ansary et al. (2022). Furthermore, Pointner et al. (2026) similarly identified *p-*coumaric acid as the most abundant polyphenol in black cumin seed oil. Herbacetin and secoisolariciresinol were also detected in the oil and remained essentially constant during green extraction, at about 11 mg/kg and about 5 mg/kg, respectively. Taken together, the polyphenol results indicate that green extraction had little effect on the overall phenolic load of the oil, but they also confirm that the oil already possessed a rich native phenolic profile before optimization.

Although Mazaheri, Torbati, Azadmard-Damirchi and Savage, 2019a, Mazaheri, Torbati, Azadmard-Damirchi and Savage, 2019b reported that roasting black cumin seeds may increase the yield of total phenols in the oil, no significant roasting-related increase in TPC, measured by Folin-Ciocalteu assay (Fig. 5), was observed in the present study. Instead, TPC showed a modest increase after 30 min of green extraction, indicating only a limited improvement in phenolic extractability at this time point. Since TPC returned to the initial level in R-BCO-90, seed roasting appears to have had little overall impact on phenolic transfer during green extraction, and no further polyphenol analyses were therefore conducted for R-BCO.

### 3.3 Oxidation markers and hydrolytic stability of unroasted oil

The photometric determination of CD is shown in Fig. 4A. It provides information on primary lipid oxidation processes and showed that green extraction affected this parameter in BCO. The diene concentration decreased slightly but significantly from 0.05 ± 0.002 μmol/mL in BCO-0 to 0.05 ± 0.001 μmol/mL after 30 min and 0.04 ± 0.002 μmol/mL after 90 min. The diene content of the oil may be related to its fatty acid composition, because linoleic acid is a major precursor in the formation of CD (Pegg, 2001; Pignitter et al., 2014). However, the slight decline during green extraction suggests that the process did not promote diene accumulation and may even have reduced these primary oxidation-related species to a small extent.

**Fig. 4:**
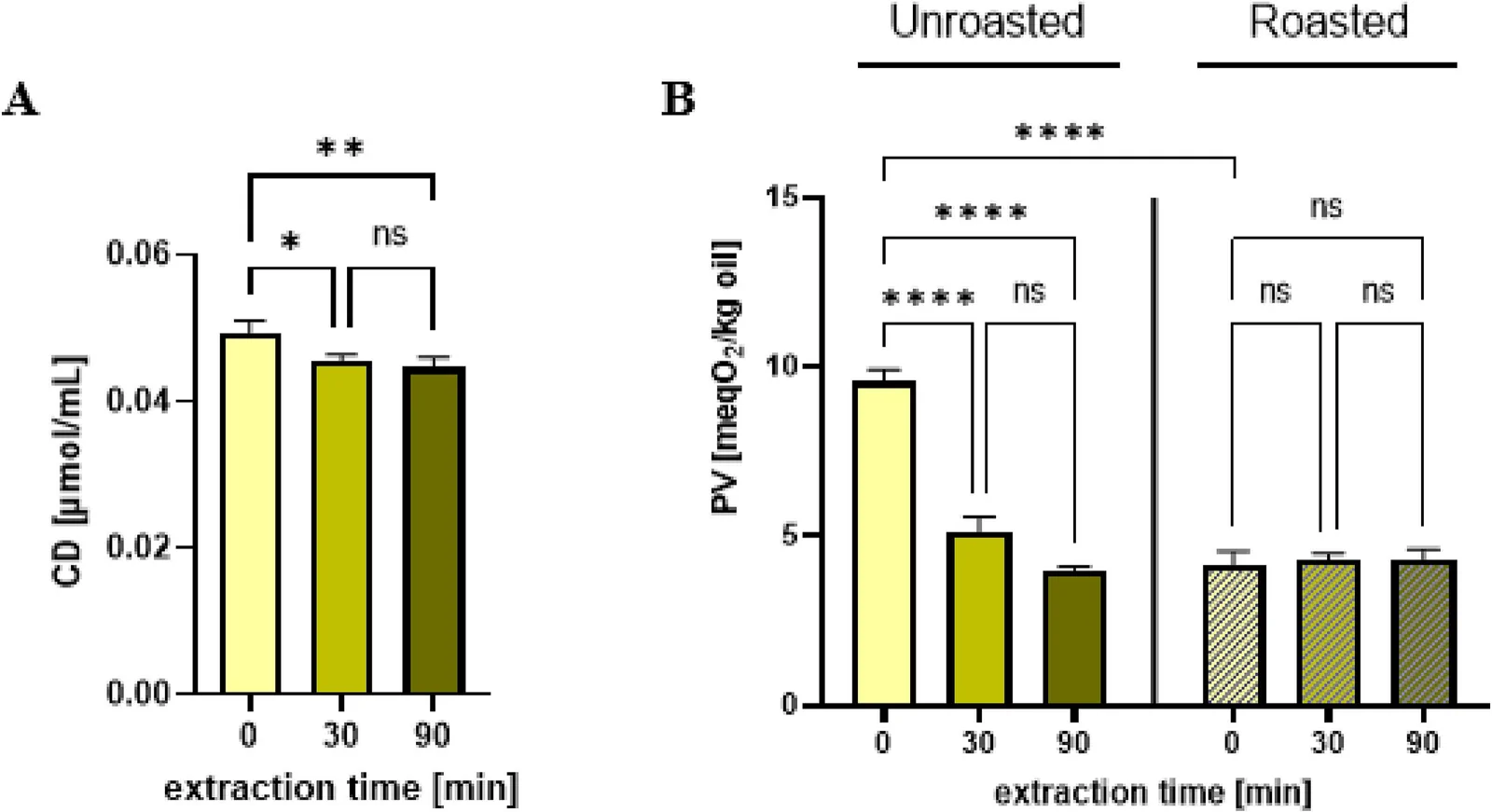
Analysis of primary oxidation products in oil samples. Conjugated diene (CD) content of black cumin oil from unroasted seeds (A) and peroxide value (POV) of black cumin oil from roasted and unroasted seeds (B), determined spectrophotometrically following green extraction for 0, 30, and 90 min. Data are expressed as mean ± standard deviation (*n* = 3). Statistical significance was assessed via one-way ANOVA followed by Tukey's post-hoc test for CD and Šídák's post-hoc test for POV: ns, non-significant; *p < 0.05; ** *p* < 0.01, **** *p* < 0.0001. (For interpretation of the references to colour in this figure legend, the reader is referred to the web version of this article.)

A different pattern was observed for acid value and free fatty acids, which proved to be the most critical quality parameters of the unroasted oil (Fig. 6). High levels of free fatty acids can negatively affect oil quality and accelerate oxidation as well as sensory deterioration. According to international food quality standards, the acid value of unrefined edible oils should not exceed 4 mg KOH/g oil (Ashrafi et al., 2023; Codex Alimentarius Commission, 1999). In the investigated oil, however, the acid value already amounted to 8.50 ± 0.30 mg KOH/g in BCO-0 and therefore exceeded this limit even before green extraction. After 30 min of green extraction, the acid value increased significantly to 22.55 ± 1.10 mg KOH/g, and after 90 min it rose further to 24.67 ± 0.27 mg KOH/g. The corresponding free fatty acid content increased from 4.24 ± 0.15% in BCO-0 to 12.31 ± 0.13% in BCO-90.

**Fig. 6:**
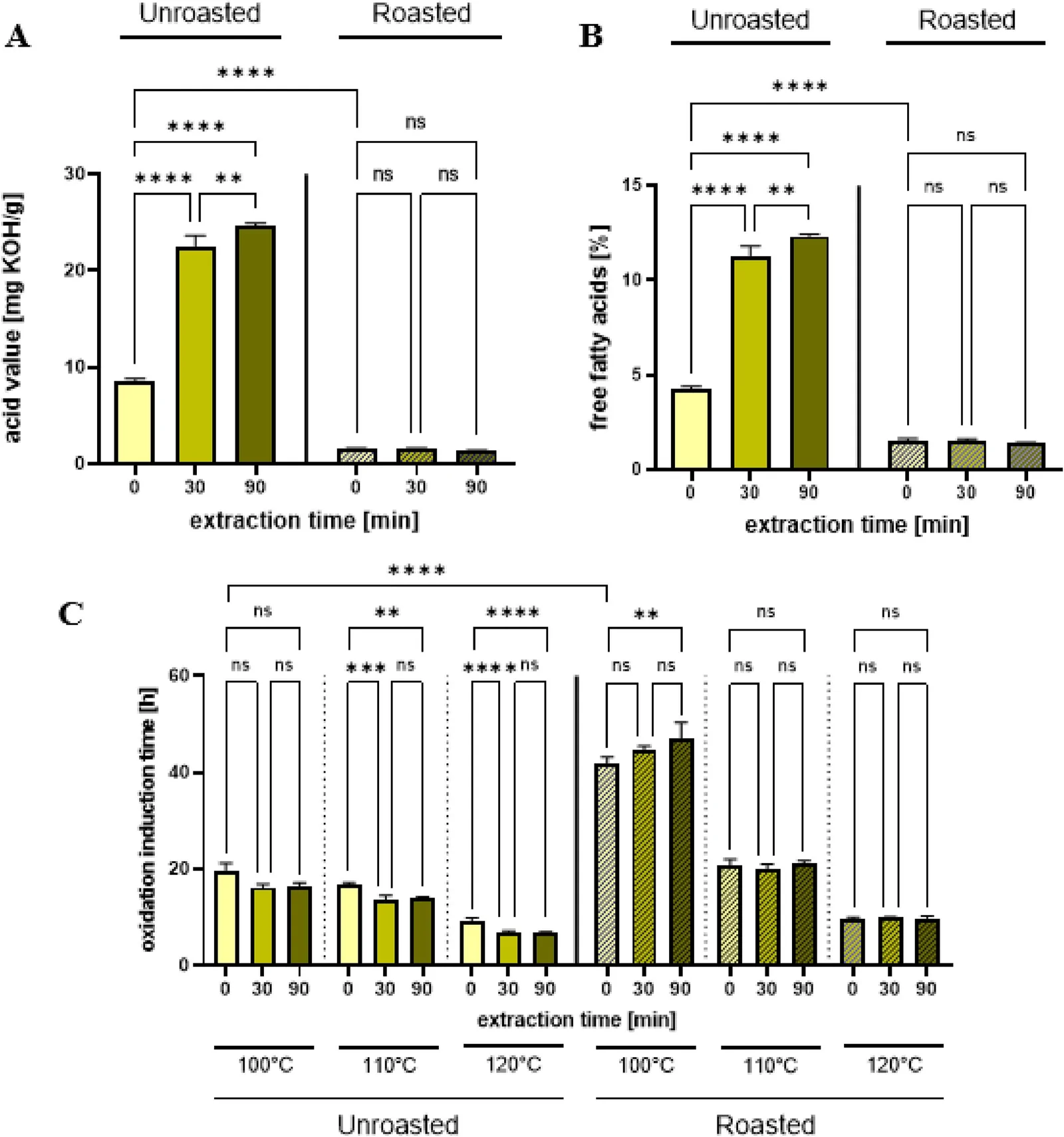
Influence of green extraction (0, 30, and 90 min) on the (A) acid value (AV), (B) free fatty acid (FFA) levels and OIT (C) of oils derived from both roasted and unroasted seeds, determined via titrimetric analysis and by Rancimat analysis at 100 °C, 110 °C, and 120 °C. Data are expressed as mean ± standard deviation (*n* = 3). Statistical significance was assessed via one-way ANOVA followed by Šídák's post-hoc test: ns, non-significant; ** *p* < 0.01; *** *p* < 0.001, *p* < 0.0001. (For interpretation of the references to colour in this figure legend, the reader is referred to the web version of this article.)

These data show that although green extraction improved several antioxidant-related parameters, it strongly impaired the hydrolytic stability of the oil from unroasted seeds. Lipase and lipoxygenase activities from enzymes are present in black cumin seeds and in the press cake. Therefore, it can be hypothesized that these enzymes may have mediated the increase in free fatty acids and acid value. The extraction procedure involved remixing the oil with press cake, which could facilitate the transfer of active enzymes into the oil phase and thereby intensify lipid hydrolysis. Data from literature supports the relevance of this issue for BCO in general, as Dąbrowski et al. (2024) reported very high acid values from 19.7 ± 0.5 mg KOH/g up to 52.3 ± 0.8 mg KOH/g, and Szydłowska-Czerniak et al. (2022) reported values from 6.40 ± 0.06 mg KOH/g to 22.02 ± 0.08 mg KOH/g. These studies concluded that none of the analyzed BCOs fulfilled the applicable limits for oxidative and hydrolytic stability, which could potentially create health risks for consumers (Dąbrowski et al., 2024; Szydłowska-Czerniak et al., 2022).

The peroxide value, presented in Fig. 4B, showed yet another response to green extraction and, unlike the acid value, moved in a favorable direction. According to the Codex Alimentarius, the peroxide value of unrefined edible oils should not exceed 15 meq O₂/kg, although this limit does not explicitly apply to BCO because its naturally high essential oil content can lead to higher peroxide values. The initial peroxide value was 9.56 ± 0.36 meq O_2_/kg. Green extraction significantly reduced this parameter, with the value falling to about half after 30 min and decreasing further to 3.98 ± 0.13 meq O_2_/kg after 90 min. This trend agrees with the effects described by Pointner et al. (2025) for linseed oil, where green extraction also significantly reduced the peroxide value.  However, the peroxide value may also be influenced by hydroperoxide decomposition, matrix effects, or partitioning during the green extraction process, and therefore does not solely reflect antioxidant accumulation. Secondary oxidation markers measured by SPME-GC–MS (Fig. 7B) supported the finding from the determination of CD and peroxide value. Hexanal remained unchanged during green extraction, whereas hexanol decreased. Together with the primary oxidation data, these results indicate that green extraction did not worsen, and may even improve, the oxidative status of the oil.

**Fig. 7:**
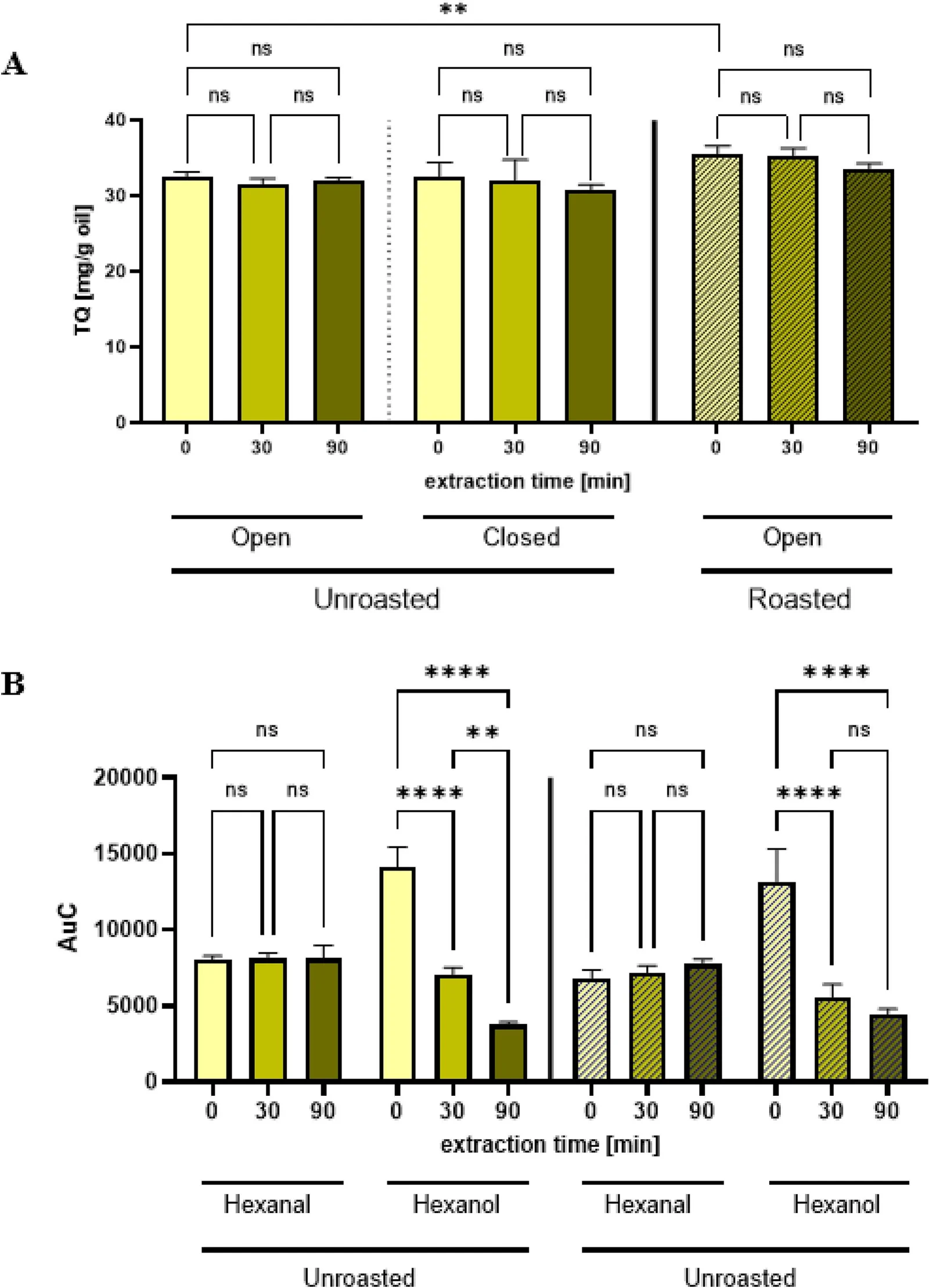
GC–MS analysis of black cumin oil from roasted and unroasted seeds as a function of green extraction time (0, 30, and 90 min). Quantification of thymoquinone (TQ) in black cumin oil (open and closed systems) (A), as well as semi quantitative determination of hexanal and hexanol comparing area under the curve (AuC) (B). Data are expressed as mean ± standard deviation (*n* = 3). Statistical significance was assessed via one-way ANOVA followed by Šídák's post-hoc test: ns, non-significant; ** *p* < 0.01, **** *p* < 0.0001. (For interpretation of the references to colour in this figure legend, the reader is referred to the web version of this article.)

This apparently paradoxical combination of falling peroxide values and rising acid values may be explained by residual antioxidant substances still present in the press cake contributing to neutralization of free radicals and thereby lowering the peroxide value during green extraction. A high DPPH radical-scavenging capacity was reported in black cumin press cake, which was linked to high concentrations of remaining phenolic compounds of up to 4260 mg GAE/kg (Rabiej-Kozioł & Szydłowska-Czerniak, 2024; Szydłowska-Czerniak et al., 2022). At the same time, the present results indicate that the extraction conditions were not sufficient to transfer these compounds into the oil to a major extent, because the total polyphenol concentration of the oil itself did not increase significantly. On the other hand, this study showed that the green extraction process successfully transferred other antioxidants like chlorophyll, carotenoids and α-tocopherol into the oil. Further analysis of the press cake would be necessary for a more precise evaluation of these mechanisms.

### 3.4 Effects of seed roasting on quality and oxidative stability

Because the hydrolytic stability of the oil from unroasted seeds (BCO) was poor and worsened during green extraction, the study investigated whether roasting the seeds before pressing could stabilize the system (R-BCO). The rationale was that heat treatment might inactivate lipases and lipoxygenases, which had been proposed as major drivers of free fatty acid formation. Mazaheri et al. (2019b) roasted black cumin seeds at 180 °C for 8 min and observed a significant decrease in acid value to below the regulatory limit (Mazaheri et al., 2019b). The same roasting approach was therefore applied in the present work.

The pronounced effect on the acid value in BCO is shown in Fig. 6. After roasting and subsequent cold pressing, the acid value of the oil decreased to 3.09 ± 0.18 mg KOH/g in R-BCO-0 (compared to 8.50 ± 0.30 mg KOH/g in BCO-0). This value is below the Codex Alimentarius limit of 4 mg KOH/g and means that R-BCO meets the Codex acid-value criterion for unrefined edible oils (Codex Alimentarius Commission, 1999). The free fatty acid content calculated from the acid value was 1.54 ± 0.09% in R-BCO-0. In contrast to the strong increase observed in BCO, green extraction of R-BCO did not cause any significant deterioration of this parameter, and the free fatty acid contents remained constant at 1.54 ± 0.09% in R-BCO-0, 1.53 ± 0.09% in R-BCO-30, and 1.38 ± 0.06% in R-BCO-90. These findings support the hypothesis that roasting effectively suppressed the enzymatic processes responsible for hydrolysis.

Roasting also had a beneficial effect on the peroxide value of the oil (Fig. 4). The initial peroxide value in the roasted sample R-BCO-0 was only 4.13 ± 0.43 meq O_2_/kg, compared with 9.56 ± 0.36 meq O_2_/kg in the unroasted oil (BCO-0). This represents a clear reduction due to the heat pretreatment of the seeds. Green extraction of the roasted oil did not produce a further significant decrease. Data from literature shows that the effect of roasting on peroxide value can be inconsistent in BCO, because Mazaheri et al. (2019b) reported a slight increase at 180 °C, whereas Suri et al. (2019) observed that peroxide value decreased significantly at 180 °C after an initial increase at 160 °C. The present data clearly align with the latter pattern and indicate that roasting at 180 °C can improve primary oxidation status under the conditions used here. Secondary oxidation markers in R-BCO showed a similar trend to BCO (Fig. 7B). Hexanal remained stable at all time points, whereas hexanol decreased significantly in both R-BCO-30 and R-BCO-90; no difference was observed between BCO-0 and R-BCO-0. Overall, these data suggest that combining seed roasting with green extraction improves both primary and secondary oxidation status.

The Rancimat measurements further demonstrated that roasting substantially improved oxidative stability (Fig. 6C). For the oil produced from unroasted seeds, the oxidative induction time at 100 °C was 19.42 ± 1.80 h in BCO-0. Comparable induction times at 100 °C were reported by Cheikh-Rouhou et al. (2007), who determined OIT values of 12.0 ± 0.05 h and 50.33 ± 2.48 h in two different BCOs. Green extraction did not increase this value of BCO and the OIT of the oil even decreased significantly at 110 °C and 120 °C. This decline may be a consequence of the extraction procedure itself, because lipolytic enzymes transferred from the press cake may be responsible for the increase in acid value and peroxide-related deterioration, thereby shortening oxidative stability. This assessment is supported by Frega et al. (1999) who consistently observed a reduction in oxidative induction time with increasing acid values in edible oils (Frega et al., 1999). Despite the lower induction times under accelerated oxidation conditions, Arrhenius-based shelf-life predictions at 25 °C indicated an approximately constant stability of up to two months for the unroasted samples.

R-BCO behaved differently. Significantly longer induction times at 100 °C were measured after prior roasting of the black cumin seeds. The OIT increased from 19.42 ± 1.80 h in BCO-0 to 41.77 ± 1.40 h in R-BCO-0. This may be explained by inactivation of lipases and lipoxygenases and the resulting reduction in rapidly oxidizable free fatty acids and hydroperoxides (Mazaheri et al., 2019b). Roasting before pressing can also improve oil stability because it may generate Maillard reaction products (MRPs), such as melanoidins and pyrrole compounds, that act as antioxidants and protect unsaturated lipids (Różańska et al., 2019; Shrestha & De Meulenaer, 2014; Suri et al., 2019). However, the lack of a significant difference in browning index between BCO-0 and R-BCO-0 is an indication that MRPs contributed only modestly to the improved OIT of R-BCO-0, with other roasting-induced changes probably playing a larger role. In the roasted approach, green extraction was no longer detrimental; instead, R-BCO-90 showed a significant increase in OIT from 41.77 ± 1.40 h in R-BCO-0 to 46.86 ± 3.51 h in R-BCO-90. The Arrhenius-based storage predictions at 25 °C reflected this improvement, indicating an 85% increase in the predicted shelf life of R-BCO-90 relative to R-BCO-0 (Table S27). The Arrhenius model is based on measurements at only three temperatures and is strongly influenced by the slope of the regression curve. Accordingly, the resulting shelf-life predictions should be regarded strictly as estimates from an accelerated oxidation test, rather than as validated real-time shelf-life values. Even with this limitation, the data clearly shows that green extraction had a positive impact on OIT after the seed material had first been stabilized by roasting. Notably, the combined impact of roasting and subsequent green extraction yielded an OIT increase of **+**27.4 h. This improvement surpassed the simple additive effects of roasting and green extraction performed with oil from unroasted seeds (**+**19.2 h), indicating a clear synergistic effect. From a statistical perspective, synergism between the two factors can be interpreted as an interaction, which occurs when the combined effect of two factors cannot be predicted from their individual effects alone. In this context, synergism is present when the joint effect exceeds the effect expected under the null hypothesis of non-interaction (Anastasio et al., 2015). Statistically, such non-additive behavior can be assessed using a two-way ANOVA, where a significant interaction term indicates that the effect of one factor depends on the level of the other (Alin & Kurt, 2006). In the present study, two-way ANOVA revealed a significant roasting × optimization interaction regarding OIT (*p* = 0.003), indicating that the effect of optimization was contingent on roasting status. This non-additive response suggests that roasting modified the effect of optimization rather than acting as an independent factor, thereby providing statistical evidence of synergism between roasting and optimization on OIT. The full two-way ANOVA table, including SS, DF, MS, F, p value, percent total variation, and partial eta squared, is reported in Table S6. Tukey post-hoc comparisons with 95% confidence intervals are provided in Table S7. Evaluation of the roasting × optimization interaction also revealed a significant interaction for carotenoids (*p* = 0.0008), chlorophylls (*p* = 0.0072), acid value (*p* < 0.0001), free fatty acids (*p* < 0.0001) and peroxide value (*p* < 0.0001), while the interaction for tocopherols, total phenolic content, thymoquinone, hexanal and hexanol was not significant (Tables S11-S26). Because conjugated dienes, ABTS and FRAP were determined only for oils from unroasted seeds, these outcomes were only analyzed across extraction times by one-way ANOVA and were not included in the factorial interaction analysis. This study demonstrated the efficacy of green extraction in facilitating the transfer of chlorophylls, carotenoids, and tocopherols into the BCO matrix. A similar enrichment occurred in the BCO derived from roasted seeds, contributing to the observed increase in OIT following the green extraction process.

### 3.5 Thymoquinone and extraction mode

Results of the GC–MS analysis are shown in Fig. 7. TQ was identified as a major bioactive marker of BCO and showed that its concentration was high already in the untreated sample. The TQ content in BCO-0 was 32.56 ± 0.61 mg/g. This concentration is notably higher than those reported in existing literature; for instance, Lutterodt et al., 2010 found TQ levels ranging from 3.48 to 8.73 mg/g across six distinct BCO varieties, while Alkhatib et al. (2020) reported values between 1.4 and 18.8 mg/g. Despite the high native content, green extraction for 30 or 90 min yielded no statistically significant alterations in the TQ concentration of the oil.

Because TQ is a volatile compound, the study additionally examined whether losses might have occurred during green extraction in the open system. To address this question, an additional green extraction was carried out in a closed system under exclusion of oxygen, and TQ was quantified again by GC–MS. Since the open and closed systems used different mixing devices, the comparison should be interpreted qualitatively rather than as a strict assessment of identical hydrodynamic conditions. The results showed that the method of mixing with the press cake had no significant influence on TQ concentration in the oil. Starting from 32.56 ± 0.61 mg/g in C-BCO-0, the values remained largely constant at 32.01 ± 2.78 mg/g in C-BCO-30 and 30.68 ± 0.77 mg/g in C-BCO-90. These findings are consistent with the observations of Neunert et al. (2025) who reported that TQ concentrations decreased significantly only after approximately three days in open systems, whereas no substantial degradation was observed over 21 days in closed samples. On that basis, the present work concludes that the short-term mixing during green extraction was too brief to cause a measurable TQ loss.

Roasting had a clearer effect on TQ than green extraction itself. In the roasting experiment, the TQ concentration in the oil increased significantly from 32.66 ± 0.60 mg/g in BCO-0 to 35.57 ± 1.05 mg/g in R-BCO-0. However, a subsequent green extraction of the R-BCO did not lead to any further significant increase. This result fits the broader pattern observed throughout the study. Seed roasting was the decisive pretreatment for improving the technological and oxidative properties of BCO, whereas green extraction mainly modified selected antioxidant and pigment-related parameters and was particularly advantageous when applied to already stabilized material. Because headspace quantification reflects equilibrium partitioning, its response is matrix dependent and cannot be assumed to be universally transferable across oil matrices without validation (Ai, 1997). For this reason, the key validation parameters are presented in the Supplementary Material (Tables S9 and S10).

## 4 Conclusions

This study shows that green extraction with black cumin press cake can improve the antioxidant profile and oxidative stability of cold-pressed black cumin oil, especially when applied after roasting. The combined process is attractive from an industrial perspective because it uses food-grade materials already present in the production chain and avoids organic solvents, offering a simple route to valorize press cake and upgrade oil quality. At the same time, the approach does not resolve the high acidity of the oil when unroasted cake is used, indicating that hydrolytic quality remains a limitation. Roasting effectively reduced acid values and enhanced the stability of the final oil, suggesting that thermal pretreatment is an important step for practical application.

Overall, roasting followed by green extraction appears to be a promising post-pressing strategy for improving the quality of black cumin oil. Before industrial implementation, however, process optimization, sensory evaluation, and pilot-scale validation are needed to confirm product quality, consumer acceptance, and scalability.

## Declaration of generative AI and AI-assisted technologies in the manuscript preparation process

During the preparation of this work, the authors used ChatGPT (GPT-5, OpenAI, San Francisco, USA) to improve the structure and language of the manuscript. *DeepL.com* (v. 3.5, DeepL SE, Cologne, Germany) was used for language refinement and translation of sections originally drafted in German. After using these tools, the authors carefully reviewed and edited all content to ensure accuracy and take full responsibility for the integrity and originality of the published work.

## CRediT authorship contribution statement

**Michael Zeugswetter:** Writing – original draft, Visualization, Methodology, Investigation, Formal analysis, Data curation. **Tobias Pointner:** Writing – review & editing, Visualization, Methodology, Investigation, Formal analysis, Data curation. **Lisa Simettinger:** Writing – review & editing, Visualization, Methodology, Investigation, Formal analysis, Data curation. **Marc Pignitter:** Writing – review & editing, Visualization, Supervision, Resources, Project administration, Funding acquisition, Conceptualization.

## Declaration of competing interest

The authors declare that they have no known competing financial interests or personal relationships that could have appeared to influence the work reported in this paper.
