# Supplementary material for: Synergistic effect of seed roasting and green extraction on oxidative stability in black cumin oil
Source: Food Chem X. 2026 Oct 22;40:104671. doi: 10.1016/j.fochx.2026.104671 (PMC13720460; doi:10.1016/j.fochx.2026.104671)
Supplement: Supplementary file 1 [file mmc1.docx]

**Synergistic Effect of Seed Roasting and Green Extraction on Oxidative Stability in Black Cumin Oil**

Michael Zeugswetter^a,b *^, Tobias Pointner^a,b *^, Lisa Simettinger^a^, Marc Pignitter^a,c^

^a^ Institute of Physiological Chemistry, Faculty of Chemistry, University of Vienna, 1090 Vienna, Austria

^b^ Vienna Doctoral School in Chemistry (DoSChem), University of Vienna, Währinger Str. 42, 1090 Vienna, Austria

^c^ Corresponding author

* Joint first authors

Michael ZEUGSWETTER: [michael.zeugswetter@univie.ac.at](mailto:michael.zeugswetter@univie.ac.at)

Tobias POINTNER: [tobias.pointner@univie.ac.at](mailto:tobias.pointner@univie.ac.at)

Lisa SIMETTINGER: [a11932031@unet.univie.ac.at](mailto:a11932031@unet.univie.ac.at)

Corresponding author: Marc PIGNITTER: [marc.pignitter@univie.ac.at](mailto:marc.pignitter@univie.ac.at); Phone: +43 1 4277 – 70621

# Supplementary Information

**Table S1:** *Calibration parameters for the quantification of α-, γ-, and δ-tocopherol in black cumin oil via HPLC-UV analysis and coefficient of determination (R²).*

| Standard | Formula | R^2^ [%] |
| --- | --- | --- |
| alpha-tocopherol | y = 31116x - 14728 | 99.99 |
| gamma-tocopherol | y = 38852x - 17129 | 99.99 |
| delta-tocopherol | y = 39875x - 26950 | 99.99 |

**Table S2:** *Calibration parameters for the quantification of thymoquinone in black cumin oil via SPME‑GC‑MS analysis and coefficient of determination (R²).*

| Standard | Formula | R^2^ [%] |
| --- | --- | --- |
| Thymoquinone | y = 187693x - 195658 | 99.66 |

**Table S3**: Calibration parameters for the six representative standards used in the quantification of phenolic compounds via LC-MS/MS, including the coefficient of determination (R^2^), limit of detection (LOD, S/N = 3), and limit of quantification (LOQ, S/N = 10).

| Standard | Formula | R^2^ [%] | LOD [µg/mL] | LOQ [µg/mL] |
| --- | --- | --- | --- | --- |
| Caffeic acid | y = 11738x + 12687 | 98.18 | 0.13 | 0.42 |
| p-coumaric acid | y = 19998x + 18570 | 98.54 | 0.33 | 1.09 |
| Secoisolariciresinol | y = 15653x + 2854.7 | 99.40 | 0.61 | 2.03 |
| Gallic acid | y = 3756.2x + 1201.4 | 95.55 | 0.69 | 2.30 |
| Ferulic acid | y = 44021x + 2312.5 | 99.62 | 1.18 | 3.93 |
| Quercetin | y = 3280.9x - 4318.8 | 97.8 | 0.84 | 2.80 |

**Table S4.** Comparison of the phenolic compounds identified across the various black cumin oil samples (n = 3), as determined by LC-MS/MS analysis.

|  | **BCO-0** | | | **BCO-30** | | | **BCO-90** | | |
| --- | --- | --- | --- | --- | --- | --- | --- | --- | --- |
| **Phenolic compound [mg/kg]** | **Avg** | **SD** | **RSD%** | **Avg** | **SD** | **RSD%** | **Avg** | **SD** | **RSD%** |
| Herbacetin | 11.84 | 1.49 | 12.59 | 11.15 | 1.05 | 9.42 | 11.05 | 2.57 | 23.27 |
| Secoisolariciresinol | 5.56 | 0.41 | 7.37 | 4.15 | 0.51 | 12.29 | 5.30 | 0.54 | 10.19 |
| Caffeic acid | 0.84 | 0.29 | 34.52 | 0.68 | 0.21 | 30.88 | 1.00 | 0.31 | 31.54 |
| Cinnamic acid | 2.63 | 0.20 | 7.60 | 2.30 | 0.91 | 39.57 | 1.66 | 0.28 | 17.07 |
| *ρ*-Cumaric acid | 67.29 | 12.20 | 18.13 | 62.88 | 15.89 | 25.27 | 76.14 | 19.87 | 26.09 |
| Quinic acid | 1.49 | 0.53 | 35.57 | 2.08 | 0.30 | 14.57 | 2.66 | 0.51 | 19.32 |

**Table S5.** MRM transitions of polyphenolic substances analyzed via targeted LC-MS analysis.

| **Substances** | Polyphenol Class | m/z Precursor + fragments |
| --- | --- | --- |
| Chalcone | Flavonoids | 209.1-131.1-103.1 |
| (+)-Catechin | Flavan-3-ols | 289.1-245.1-123.0-109.0 |
| Epicatechin | Flavan-3-ols | 289.1-245.1-125.0-109.0 |
| Gallocatechol | Flavan-3-ols | 305.1-303.1-137.0-125.0 |
| Amentoflavone | Flavones | 539.1-497.1-403.0-377.1 |
| Herbacetin | Flavones | 303.0-169.0-133.0-121.0 |
| Apigenin | Flavonoids | 269.0-151.0-117.0-107.0 |
| Astragalin | Flavonoids | 447.4-284.0-255.0-227.0 |
| Eriodictyol | Flavonoids | 287.1-255.0-151.0-135.0 |
| Hesperetin | Flavonoids | 303.3-302.0-177.0-153.0 |
| Hesperidin | Flavonoids | 609.2-303.1-302.1-301.1 |
| Isorhamnetin | Flavonoids | 315.1-300.0-151.0-107.0 |
| Isorhamnetin 3-rutinoside | Flavonoids | 625.2-318.1-302.1 |
| Kaempferol | Flavonoids | 285.1-267.0-257.0-229.0 |
| Kaempferol-3-O-arabinoside | Flavonoids | 417.1-284.0-255.0-227.0 |
| Kaempferol-3-O-glucoside (Astragalin) | Flavonoids | 447.1-284.0-255.0 |
| Kaempferol-3-O-glucuronic acid | Flavonoids | 461.1-285.0-284.0-229.1 |
| Luteolin | Flavonoids | 285.0-151.0-133.0-107.0 |
| Luteolin-7-O-glucoside | Flavonoids | 447.1-285.0-284.0-151.0 |
| Naringenin | Flavonoids | 271.2-270.0-151.0-119.0 |
| Naringin | Flavonoids | 579.2-459.1-271.1-151.0 |
| Quercetin | Flavonoids | 301.0-121.0-93.0 |
| Quercetin-3-O-glucoronic acid | Flavonoids | 477.1-301.0-151.0-107.0 |
| Quercetin-3-O-glucoside | Flavonoids | 463.4-301.0-300.0-271.0 |
| Rutin | Flavonoids | 609.2-300.0-299.0-271.0 |
| Lariciresinol | Lignans | 359.2-330.1-329.1-160.1 |
| Matairesinol | Lignans | 357.1-137.1-122.0-83.0 |
| Pinoresinol | Lignans | 357.1-163.0-151.0-136.0 |
| Secoisolariciresinol | Lignans | 363.2-327.2-137.1 |
| Secoisolariciresinol diglucoside | Lignans | 685.3-524.2-523.2-361.2 |
| 1.3-Dicaffeoylquinic acid | Phenolic Acids | 515.0-353.0-191.0-179.0 |
| 3.4-Dimethoxy-cinnamic acid | Phenolic Acids | 207.1-119.1-103.1 |
| 4-hydroxy-benzoic acid | Phenolic Acids | 137.1-119.1-93.0-59.2 |
| 4-O-Caffeoylquinic acid | Phenolic Acids | 353.1-191.0-179.0-173.0 |
| Caffeic acid | Phenolic Acids | 179.0-135.0-107.1-97.0 |
| Caffeic acid hexoside | Phenolic Acids | 341.1-180.0-179.0-135.0 |
| Chlorogenic acid | Phenolic Acids | 353.1-191.2-179.0-173.2 |
| Cinnamic acid | Phenolic Acids | 149.1-131.1-126.0-103.1 |
| Coumaric acid hexoside | Phenolic Acids | 325.1-205.0-145.0-117.0 |
| Ellagic acid | Phenolic Acids | 303.0-285.0-257.0-229.0-80.1 |
| Ferulic acid | Phenolic Acids | 195.0-177.0-117.0-89.0 |
| Gallic acid | Phenolic Acids | 169.0-125.0-96.9-79.1 |
| Gallic acid hexoside | Phenolic Acids | 331.1-170.0-169.0-125.0 |
| Isoferulic acid | Phenolic Acids | 193.0-178.3-133.8-95.1 |
| p-coumaric acid | Phenolic Acids | 165.1-147.0-119.1-91.1 |
| p-coumaroyl-quinic acid | Phenolic Acids | 337.0-191.0-173.0-163.0 |
| p-hydroxy-benzoic acid | Phenolic Acids | 137.0-137.0-108.0-93.0 |
| Protocatechuic acid | Phenolic Acids | 153.0-109.0-108.0-81.0 |
| Quinic acid | Phenolic Acids | 191.1-93-85.0-59.0 |
| Ursolic acid | Phenolic Acids | 455.4-407.0-391.0-377.0 |
| Vanillin | Phenolic Aldehyde | 151.0-136.0-108.0-92.0 |

**Table S6**: Two-way ANOVA of the effects of seed roasting and green extraction time on the oxidative induction time of black cumin oil. OIT values for BCO-0, BCO-30, BCO-90, R‑BCO‑0, R-BCO-30, and R-BCO-90 were analyzed by two-way ANOVA.

| Source of variation | SS (Type III) | DF | MS | F | P value | % total variation | Partial eta squared |
| --- | --- | --- | --- | --- | --- | --- | --- |
| Interaction | 62.8 | 2 | 31400 | 8.760 | 0.0030 | 1.6040 | 0.5387 |
| Row factor | 5.269 | 2 | 2635 | 0.735 | 0.4960 | 0.1346 | 0.0892 |
| Column factor | 3788 | 1 | 3788000 | 1057.000 | <0.0001 | 96.7700 | 0.9860 |
| Residual | 53.77 | 15 | 3584 | — | — | — | — |

**Table S7**: Tukey’s multiple comparisons test for pairwise comparisons of OIT among black cumin oil samples subjected to roasting and green extraction treatments.

| Tukey's multiple comparisons test | Predicted (LS) mean diff. | 95,00% CI of diff. | Adjusted P Value |
| --- | --- | --- | --- |
| BCO-0 vs. R-BCO-0 | -22,35 | -27,04 to -17,65 | <0,0001 |
| BCO-0 vs. BCO-30 | 3,253 | -1,769 to 8,276 | 0,3356 |
| BCO-0 vs. R-BCO-30 | -25,25 | -29,95 to -20,55 | <0,0001 |
| BCO-0 vs. BCO-90 | 3,137 | -1,886 to 8,159 | 0,3717 |
| BCO-0 vs. R-BCO-90 | -27,44 | -32,13 to -22,74 | <0,0001 |
| R-BCO-0 vs. BCO-30 | 25,6 | 20,90 to 30,30 | <0,0001 |
| R-BCO-0 vs. R-BCO-30 | -2,908 | -7,257 to 1,442 | 0,3053 |
| R-BCO-0 vs. BCO-90 | 25,48 | 20,78 to 30,18 | <0,0001 |
| R-BCO-0 vs. R-BCO-90 | -5,09 | -9,440 to -0,7405 | 0,0176 |
| BCO-30 vs. R-BCO-30 | -28,51 | -33,20 to -23,81 | <0,0001 |
| BCO-30 vs. BCO-90 | -0,1167 | -5,139 to 4,906 | >0,9999 |
| BCO-30 vs. R-BCO-90 | -30,69 | -35,39 to -25,99 | <0,0001 |
| R-BCO-30 vs. BCO-90 | 28,39 | 23,69 to 33,09 | <0,0001 |
| R-BCO-30 vs. R-BCO-90 | -2,183 | -6,532 to 2,167 | 0,5928 |
| BCO-90 vs. R-BCO-90 | -30,57 | -35,27 to -25,87 | <0,0001 |

**Table S8**: Arrhenius regression statistics and predicted 25 °C shelf life for black cumin oils from roasted and unroasted seeds after 0, 30, and 90 min of green extraction, based on Rancimat measurements at 100–120 °C.

| **Sample** | **R^2^** | | **Ea_[kJ/mol]** | **25C_shelf_life_months** | **Shelf_life_95CI_low_months** | **Shelf_life_95CI_high_months** |
| --- | --- | --- | --- | --- | --- | --- |
| BCO-0 | | 0.8704 | 45.0773 | 1.1258 | 0.3123 | 4.0581 |
| BCO-30 | | 0.8858 | 52.3058 | 1.7071 | 0.4275 | 6.8167 |
| BCO-90 | | 0.8963 | 53.1451 | 1.8337 | 0.5858 | 5.7401 |
| R-BCO-0 | | 0.9939 | 89.4154 | 83.1768 | 53.4450 | 129.4486 |
| R-BCO-30 | | 0.9976 | 91.7762 | 104.6285 | 78.5307 | 139.3994 |
| R-BCO-90 | | 0.9940 | 95.7700 | 153.5347 | 96.1099 | 245.2702 |

**Table S9**: Validation parameters for thymoquinone quantification by HS-SPME-GC-MS in black cumin oil (BCO), including matrix effect, recovery, linearity, limit of detection (LOD), limit of quantification (LOQ), and repeatability.

| **Parameter** | **Workflow** | **Report** |
| --- | --- | --- |
| Matrix effects | Slopes of standard curves in BCO and SFO are compared | k (BCO) = 162681  k (SFO) = 182954  ME = 89% |
| Recovery | Spike native cumin oil with TQ at 3 levels (n = 3); formula:  ((measured-native)/spike concentration)*100 | See table SX |
| Linear range | Calibration curve in SFO | Range: 0-40 mg/g oil  R2 = 99.66%  LOD = 1.40 mg/g oil  LOQ = 2.59 mg/g oil |
| Repeatability | n = 3 replicate vials of BCO | RSD% (n = 3) = 0.82% |

**Table S10**: Recovery results for thymoquinone (TQ) in native cumin oil. The oil was spiked with TQ at three concentration levels (2.5, 5, and 10 mg/g oil), and recovery was calculated from the ratio of measured to added concentration.

|  | **c (TQ) [mg/g] added** | **c (TQ) [mg/g] measured** | **Recovery** |
| --- | --- | --- | --- |
|  | 2,5 | 2.93 ± 0.30 | 117% |
|  | 5 | 5,53 ± 1.35 | 111% |
|  | 10 | 9,57 ± 0.13 | 96% |

**Table S11**: Two-way ANOVA of the effects of seed roasting and green extraction time on the carotenoid content of black cumin oil. Values for BCO-0, BCO-30, BCO-90, R‑BCO‑0, R-BCO-30, and R-BCO-90 were analyzed by two-way ANOVA.

| Source of variation | SS (Type III) | DF | MS | F | P value | % total variation | Partial eta squared |
| --- | --- | --- | --- | --- | --- | --- | --- |
| Interaction | 13.970 | 2 | 6.986 | 13.86 | 0.0008 | 25.02 | 0.6978 |
| Row factor | 32.230 | 2 | 16.120 | 31.97 | <0.0001 | 57.71 | 0.8420 |
| Column factor | 3.597 | 1 | 3.597 | 7.134 | 0.0204 | 6.44 | 0.3729 |
| Residual | 6.050 | 12 | 0.5042 | — | — | — | — |

**Table S12**: Tukey’s multiple comparisons test for pairwise comparisons of carotenoid content among black cumin oil samples subjected to roasting and green extraction treatments.

| Tukey's multiple comparisons test | Predicted (LS) mean diff. | 95,00% CI of diff. | Adjusted P Value |
| --- | --- | --- | --- |
| BCO-0 vs. R-BCO-0 | 1,598 | -0,3495 to 3,545 | 0,1339 |
| BCO-0 vs. BCO-30 | -0,6958 | -2,643 to 1,252 | 0,8287 |
| BCO-0 vs. R-BCO-30 | -2,858 | -4,805 to -0,9106 | 0,0036 |
| BCO-0 vs. BCO-90 | -1,186 | -3,133 to 0,7617 | 0,3737 |
| BCO-0 vs. R-BCO-90 | -3,304 | -5,251 to -1,356 | 0,0011 |
| R-BCO-0 vs. BCO-30 | -2,294 | -4,241 to -0,3463 | 0,0183 |
| R-BCO-0 vs. R-BCO-30 | -4,456 | -6,403 to -2,508 | <0,0001 |
| R-BCO-0 vs. BCO-90 | -2,784 | -4,731 to -0,8362 | 0,0045 |
| R-BCO-0 vs. R-BCO-90 | -4,901 | -6,849 to -2,954 | <0,0001 |
| BCO-30 vs. R-BCO-30 | -2,162 | -4,109 to -0,2147 | 0,0268 |
| BCO-30 vs. BCO-90 | -0,4898 | -2,437 to 1,458 | 0,9527 |
| BCO-30 vs. R-BCO-90 | -2,608 | -4,555 to -0,6604 | 0,0074 |
| R-BCO-30 vs. BCO-90 | 1,672 | -0,2751 to 3,620 | 0,1092 |
| R-BCO-30 vs. R-BCO-90 | -0,4457 | -2,393 to 1,502 | 0,9679 |
| BCO-90 vs. R-BCO-90 | -2,118 | -4,065 to -0,1706 | 0,0305 |

**Table S13**: Two-way ANOVA of the effects of seed roasting and green extraction time on the chlorophyll content of black cumin oil. Values for BCO-0, BCO-30, BCO-90, R‑BCO‑0, R-BCO-30, and R-BCO-90 were analyzed by two-way ANOVA.

| Source of variation | SS (Type III) | DF | MS | F | P value | % total variation | Partial eta squared |
| --- | --- | --- | --- | --- | --- | --- | --- |
| Interaction | 48.230 | 2 | 24.110 | 7.991 | 0.0072 | 19.32 | 0.5924 |
| Row factor | 96.670 | 2 | 48.340 | 16.02 | 0.0006 | 38.73 | 0.7444 |
| Column factor | 60.030 | 1 | 60.030 | 19.89 | 0.0010 | 24.05 | 0.6440 |
| Residual | 33.190 | 11 | 3.017 | — | — | — | — |

**Table S14**: Tukey’s multiple comparisons test for pairwise comparisons of chlorophyll content among black cumin oil samples subjected to roasting and green extraction treatments.

| Tukey's multiple comparisons test | Predicted (LS) mean diff. | 95,00% CI of diff. | Adjusted P Value |
| --- | --- | --- | --- |
| BCO-0 vs. R-BCO-0 | 0,9229 | -3,914 to 5,760 | 0,984 |
| BCO-0 vs. BCO-30 | -1,223 | -6,060 to 3,613 | 0,948 |
| BCO-0 vs. R-BCO-30 | -7,029 | -11,87 to -2,192 | 0,0043 |
| BCO-0 vs. BCO-90 | -1,699 | -7,107 to 3,709 | 0,8829 |
| BCO-0 vs. R-BCO-90 | -8,221 | -13,06 to -3,384 | 0,0013 |
| R-BCO-0 vs. BCO-30 | -2,146 | -6,983 to 2,691 | 0,6638 |
| R-BCO-0 vs. R-BCO-30 | -7,952 | -12,79 to -3,115 | 0,0017 |
| R-BCO-0 vs. BCO-90 | -2,622 | -8,029 to 2,786 | 0,5845 |
| R-BCO-0 vs. R-BCO-90 | -9,144 | -13,98 to -4,307 | 0,0005 |
| BCO-30 vs. R-BCO-30 | -5,806 | -10,64 to -0,9685 | 0,0167 |
| BCO-30 vs. BCO-90 | -0,4752 | -5,883 to 4,933 | 0,9996 |
| BCO-30 vs. R-BCO-90 | -6,997 | -11,83 to -2,160 | 0,0045 |
| R-BCO-30 vs. BCO-90 | 5,33 | -0,07757 to 10,74 | 0,0541 |
| R-BCO-30 vs. R-BCO-90 | -1,192 | -6,029 to 3,645 | 0,9532 |
| BCO-90 vs. R-BCO-90 | -6,522 | -11,93 to -1,114 | 0,0162 |

**Table S15**: Two-way ANOVA of the effects of seed roasting and green extraction time on the tocopherol content of black cumin oil. Values for BCO-0, BCO-30, BCO-90, R‑BCO‑0, R-BCO-30, and R-BCO-90 were analyzed by two-way ANOVA.

| Source of variation | SS (Type III) | DF | MS | F | P value | % total variation | Partial eta squared |
| --- | --- | --- | --- | --- | --- | --- | --- |
| Interaction | 0.04059 | 2 | 0.02029 | 0.06114 | 0.9410 | 0.4618 | 0.0101 |
| Row factor | 3.903 | 2 | 1.952 | 5.879 | 0.0166 | 44.41 | 0.4949 |
| Column factor | 0.8615 | 1 | 0.8615 | 2.595 | 0.1332 | 9.802 | 0.1778 |
| Residual | 3.984 | 12 | 0.3320 | — | — | — | — |

**Table S16**: Tukey’s multiple comparisons test for pairwise comparisons of tocopherol content among black cumin oil samples subjected to roasting and green extraction treatments.

| Tukey's multiple comparisons test | Predicted (LS) mean diff. | 95,00% CI of diff. | Adjusted P Value |
| --- | --- | --- | --- |
| BCO-0 vs. R-BCO-0 | 0,352 | -1,228 to 1,932 | 0,9713 |
| BCO-0 vs. BCO-30 | -0,7533 | -2,333 to 0,8268 | 0,6127 |
| BCO-0 vs. R-BCO-30 | -0,1833 | -1,763 to 1,397 | 0,9985 |
| BCO-0 vs. BCO-90 | -1,157 | -2,737 to 0,4235 | 0,211 |
| BCO-0 vs. R-BCO-90 | -0,766 | -2,346 to 0,8142 | 0,5972 |
| R-BCO-0 vs. BCO-30 | -1,105 | -2,685 to 0,4748 | 0,2474 |
| R-BCO-0 vs. R-BCO-30 | -0,5353 | -2,115 to 1,045 | 0,8565 |
| R-BCO-0 vs. BCO-90 | -1,509 | -3,089 to 0,07149 | 0,0644 |
| R-BCO-0 vs. R-BCO-90 | -1,118 | -2,698 to 0,4622 | 0,238 |
| BCO-30 vs. R-BCO-30 | 0,57 | -1,010 to 2,150 | 0,8234 |
| BCO-30 vs. BCO-90 | -0,4033 | -1,983 to 1,177 | 0,9498 |
| BCO-30 vs. R-BCO-90 | -0,01267 | -1,593 to 1,567 | >0,9999 |
| R-BCO-30 vs. BCO-90 | -0,9733 | -2,553 to 0,6068 | 0,3625 |
| R-BCO-30 vs. R-BCO-90 | -0,5827 | -2,163 to 0,9975 | 0,8106 |
| BCO-90 vs. R-BCO-90 | 0,3907 | -1,189 to 1,971 | 0,9559 |

**Table S17**: Two-way ANOVA of the effects of seed roasting and green extraction time on the peroxide value of black cumin oil. Values for BCO-0, BCO-30, BCO-90, R‑BCO‑0, R-BCO-30, and R-BCO-90 were analyzed by two-way ANOVA.

| Source of variation | SS (Type III) | DF | MS | F | P value | % total variation | Partial eta squared |
| --- | --- | --- | --- | --- | --- | --- | --- |
| Interaction | 27.780 | 2 | 13.890 | 117.6 | <0.0001 | 38.99 | 0.9515 |
| Row factor | 24.590 | 2 | 12.300 | 104.1 | <0.0001 | 34.52 | 0.9455 |
| Column factor | 17.460 | 1 | 17.460 | 147.9 | <0.0001 | 24.51 | 0.9249 |
| Residual | 1.417 | 12 | 0.1181 | — | — | — | — |

**Table S18**: Tukey’s multiple comparisons test for pairwise comparisons of peroxide value among black cumin oil samples subjected to roasting and green extraction treatments.

| Tukey's multiple comparisons test | Predicted (LS) mean diff. | 95,00% CI of diff. | Adjusted P Value |
| --- | --- | --- | --- |
| BCO-0 vs. R-BCO-0 | 5,424 | 4,482 to 6,366 | <0,0001 |
| BCO-0 vs. BCO-30 | 4,467 | 3,525 to 5,410 | <0,0001 |
| BCO-0 vs. R-BCO-30 | 5,266 | 4,324 to 6,209 | <0,0001 |
| BCO-0 vs. BCO-90 | 5,579 | 4,637 to 6,521 | <0,0001 |
| BCO-0 vs. R-BCO-90 | 5,265 | 4,323 to 6,207 | <0,0001 |
| R-BCO-0 vs. BCO-30 | -0,9567 | -1,899 to -0,01432 | 0,0459 |
| R-BCO-0 vs. R-BCO-30 | -0,1577 | -1,100 to 0,7847 | 0,9918 |
| R-BCO-0 vs. BCO-90 | 0,155 | -0,7873 to 1,097 | 0,9924 |
| R-BCO-0 vs. R-BCO-90 | -0,159 | -1,101 to 0,7833 | 0,9915 |
| BCO-30 vs. R-BCO-30 | 0,799 | -0,1433 to 1,741 | 0,1157 |
| BCO-30 vs. BCO-90 | 1,112 | 0,1693 to 2,054 | 0,0181 |
| BCO-30 vs. R-BCO-90 | 0,7977 | -0,1447 to 1,740 | 0,1166 |
| R-BCO-30 vs. BCO-90 | 0,3127 | -0,6297 to 1,255 | 0,8664 |
| R-BCO-30 vs. R-BCO-90 | -0,001333 | -0,9437 to 0,9410 | >0,9999 |
| BCO-90 vs. R-BCO-90 | -0,314 | -1,256 to 0,6283 | 0,8644 |

**Table S19**: Two-way ANOVA of the effects of seed roasting and green extraction time on the total phenolic content of black cumin oil. Values for BCO-0, BCO-30, BCO-90, R‑BCO‑0, R-BCO-30, and R-BCO-90 were analyzed by two-way ANOVA.

| Source of variation | SS (Type III) | DF | MS | F | P value | % total variation | Partial eta squared |
| --- | --- | --- | --- | --- | --- | --- | --- |
| Interaction | 8,329 | 2 | 4,165 | 0.02748 | 0.9730 | 0.3775 | 0.0046 |
| Row factor | 2,781 | 2 | 1,391 | 0.009175 | 0.9909 | 0.1260 | 0.0015 |
| Column factor | 376,748 | 1 | 376,748 | 2.486 | 0.1409 | 17.07 | 0.1716 |
| Residual | 1,818,774 | 12 | 151,564 | — | — | — | — |

**Table S20**: Tukey’s multiple comparisons test for pairwise comparisons of total phenolic content among black cumin oil samples subjected to roasting and green extraction treatments.

| Tukey's multiple comparisons test | Predicted (LS) mean diff. | 95,00% CI of diff. | Adjusted P Value |
| --- | --- | --- | --- |
| BCO-0 vs. R-BCO-0 | 349,9 | -717,8 to 1418 | 0,872 |
| BCO-0 vs. BCO-30 | 42,12 | -1026 to 1110 | >0,9999 |
| BCO-0 vs. R-BCO-30 | 296,2 | -771,6 to 1364 | 0,9305 |
| BCO-0 vs. BCO-90 | 65,88 | -1002 to 1134 | >0,9999 |
| BCO-0 vs. R-BCO-90 | 330 | -737,7 to 1398 | 0,896 |
| R-BCO-0 vs. BCO-30 | -307,8 | -1376 to 759,9 | 0,9195 |
| R-BCO-0 vs. R-BCO-30 | -53,76 | -1121 to 1014 | >0,9999 |
| R-BCO-0 vs. BCO-90 | -284 | -1352 to 783,7 | 0,9409 |
| R-BCO-0 vs. R-BCO-90 | -19,94 | -1088 to 1048 | >0,9999 |
| BCO-30 vs. R-BCO-30 | 254 | -813,7 to 1322 | 0,9623 |
| BCO-30 vs. BCO-90 | 23,76 | -1044 to 1091 | >0,9999 |
| BCO-30 vs. R-BCO-90 | 287,9 | -779,9 to 1356 | 0,9377 |
| R-BCO-30 vs. BCO-90 | -230,3 | -1298 to 837,4 | 0,975 |
| R-BCO-30 vs. R-BCO-90 | 33,82 | -1034 to 1102 | >0,9999 |
| BCO-90 vs. R-BCO-90 | 264,1 | -803,6 to 1332 | 0,9558 |

**Table S21**: Two-way ANOVA of the effects of seed roasting and green extraction time on the thymoquinone content of black cumin oil. Values for BCO-0, BCO-30, BCO-90, R‑BCO‑0, R-BCO-30, and R-BCO-90 were analyzed by two-way ANOVA.

| Source of variation | SS (Type III) | DF | MS | F | P value | % total variation | Partial eta squared |
| --- | --- | --- | --- | --- | --- | --- | --- |
| Interaction | 2.879 | 2 | 1.440 | 2.132 | 0.1613 | 5.745 | 0.2621 |
| Row factor | 5.274 | 2 | 2.637 | 3.905 | 0.0494 | 10.52 | 0.3942 |
| Column factor | 33.870 | 1 | 33.870 | 50.15 | <0.0001 | 67.57 | 0.8069 |
| Residual | 8.104 | 12 | 0.6753 | — | — | — | — |

**Table S22**: Tukey’s multiple comparisons test for pairwise comparisons of thymoquinone content among black cumin oil samples subjected to roasting and green extraction treatments.

| Tukey's multiple comparisons test | Predicted (LS) mean diff. | 95,00% CI of diff. | Adjusted P Value |
| --- | --- | --- | --- |
| BCO-0 vs. R-BCO-0 | -3,008 | -5,261 to -0,7539 | 0,0076 |
| BCO-0 vs. BCO-30 | 0,9458 | -1,308 to 3,200 | 0,7214 |
| BCO-0 vs. R-BCO-30 | -2,618 | -4,872 to -0,3641 | 0,02 |
| BCO-0 vs. BCO-90 | 0,6514 | -1,602 to 2,905 | 0,9188 |
| BCO-0 vs. R-BCO-90 | -1,007 | -3,261 to 1,247 | 0,6701 |
| R-BCO-0 vs. BCO-30 | 3,953 | 1,700 to 6,207 | 0,0008 |
| R-BCO-0 vs. R-BCO-30 | 0,3898 | -1,864 to 2,644 | 0,9905 |
| R-BCO-0 vs. BCO-90 | 3,659 | 1,405 to 5,913 | 0,0016 |
| R-BCO-0 vs. R-BCO-90 | 2 | -0,2533 to 4,254 | 0,0933 |
| BCO-30 vs. R-BCO-30 | -3,564 | -5,817 to -1,310 | 0,002 |
| BCO-30 vs. BCO-90 | -0,2945 | -2,548 to 1,959 | 0,9974 |
| BCO-30 vs. R-BCO-90 | -1,953 | -4,207 to 0,3007 | 0,1046 |
| R-BCO-30 vs. BCO-90 | 3,269 | 1,015 to 5,523 | 0,004 |
| R-BCO-30 vs. R-BCO-90 | 1,611 | -0,6430 to 3,864 | 0,2298 |
| BCO-90 vs. R-BCO-90 | -1,659 | -3,912 to 0,5952 | 0,207 |

**Table S21**: Two-way ANOVA of the effects of seed roasting and green extraction time on the hexanal content of black cumin oil. Values for BCO-0, BCO-30, BCO-90, R‑BCO‑0, R-BCO-30, and R-BCO-90 were analyzed by two-way ANOVA.

| Source of variation | SS (Type III) | DF | MS | F | P value | % total variation | Partial eta squared |
| --- | --- | --- | --- | --- | --- | --- | --- |
| Interaction | 590,798 | 2 | 295,399 | 1.173 | 0.3426 | 7.785 | 0.1635 |
| Row factor | 858,527 | 2 | 429,264 | 1.704 | 0.2231 | 11.31 | 0.2212 |
| Column factor | 3,116,672 | 1 | 3,116,672 | 12.37 | 0.0042 | 41.07 | 0.5077 |
| Residual | 3,022,699 | 12 | 251,892 | — | — | — | — |

**Table S22**: Tukey’s multiple comparisons test for pairwise comparisons of hexanal content among black cumin oil samples subjected to roasting and green extraction treatments.

| Tukey's multiple comparisons test | Predicted (LS) mean diff. | 95,00% CI of diff. | Adjusted P Value |
| --- | --- | --- | --- |
| BCO-0 vs. R-BCO-0 | 1238 | -138,5 to 2614 | 0,0875 |
| BCO-0 vs. BCO-30 | -79,33 | -1456 to 1297 | >0,9999 |
| BCO-0 vs. R-BCO-30 | 821 | -555,5 to 2197 | 0,3938 |
| BCO-0 vs. BCO-90 | -94,67 | -1471 to 1282 | 0,9999 |
| BCO-0 vs. R-BCO-90 | 263,7 | -1113 to 1640 | 0,985 |
| R-BCO-0 vs. BCO-30 | -1317 | -2694 to 59,12 | 0,0636 |
| R-BCO-0 vs. R-BCO-30 | -417 | -1793 to 959,5 | 0,9033 |
| R-BCO-0 vs. BCO-90 | -1333 | -2709 to 43,79 | 0,0598 |
| R-BCO-0 vs. R-BCO-90 | -974,3 | -2351 to 402,1 | 0,2376 |
| BCO-30 vs. R-BCO-30 | 900,3 | -476,1 to 2277 | 0,306 |
| BCO-30 vs. BCO-90 | -15,33 | -1392 to 1361 | >0,9999 |
| BCO-30 vs. R-BCO-90 | 343 | -1033 to 1719 | 0,9544 |
| R-BCO-30 vs. BCO-90 | -915,7 | -2292 to 460,8 | 0,2908 |
| R-BCO-30 vs. R-BCO-90 | -557,3 | -1934 to 819,1 | 0,7484 |
| BCO-90 vs. R-BCO-90 | 358,3 | -1018 to 1735 | 0,9457 |

**Table S21**: Two-way ANOVA of the effects of seed roasting and green extraction time on the hexanol content of black cumin oil. Values for BCO-0, BCO-30, BCO-90, R‑BCO‑0, R-BCO-30, and R-BCO-90 were analyzed by two-way ANOVA.

| Source of variation | SS (Type III) | DF | MS | F | P value | % total variation | Partial eta squared |
| --- | --- | --- | --- | --- | --- | --- | --- |
| Interaction | 3,861,768 | 2 | 1,930,884 | 1.538 | 0.2543 | 1.211 | 0.2041 |
| Row factor | 298,418,988 | 2 | 149,209,494 | 118.9 | <0.0001 | 93.55 | 0.9520 |
| Column factor | 1,644,089 | 1 | 1,644,089 | 1.310 | 0.2748 | 0.5154 | 0.0984 |
| Residual | 15,062,927 | 12 | 1,255,244 | — | — | — | — |

**Table S22**: Tukey’s multiple comparisons test for pairwise comparisons of hexanol content among black cumin oil samples subjected to roasting and green extraction treatments.

| Tukey's multiple comparisons test | Predicted (LS) mean diff. | 95,00% CI of diff. | Adjusted P Value |
| --- | --- | --- | --- |
| BCO-0 vs. R-BCO-0 | 1022 | -2051 to 4094 | 0,8654 |
| BCO-0 vs. BCO-30 | 7124 | 4051 to 10197 | <0,0001 |
| BCO-0 vs. R-BCO-30 | 8595 | 5523 to 11668 | <0,0001 |
| BCO-0 vs. BCO-90 | 10365 | 7292 to 13437 | <0,0001 |
| BCO-0 vs. R-BCO-90 | 9685 | 6612 to 12758 | <0,0001 |
| R-BCO-0 vs. BCO-30 | 6102 | 3030 to 9175 | 0,0003 |
| R-BCO-0 vs. R-BCO-30 | 7574 | 4501 to 10646 | <0,0001 |
| R-BCO-0 vs. BCO-90 | 9343 | 6270 to 12416 | <0,0001 |
| R-BCO-0 vs. R-BCO-90 | 8663 | 5591 to 11736 | <0,0001 |
| BCO-30 vs. R-BCO-30 | 1471 | -1601 to 4544 | 0,6086 |
| BCO-30 vs. BCO-90 | 3241 | 168,0 to 6313 | 0,0367 |
| BCO-30 vs. R-BCO-90 | 2561 | -511,7 to 5634 | 0,125 |
| R-BCO-30 vs. BCO-90 | 1769 | -1303 to 4842 | 0,4286 |
| R-BCO-30 vs. R-BCO-90 | 1090 | -1983 to 4162 | 0,8329 |
| BCO-90 vs. R-BCO-90 | -679,7 | -3752 to 2393 | 0,9721 |

**Table S23**: Two-way ANOVA of the effects of seed roasting and green extraction time on the acid value of black cumin oil. Values for BCO-0, BCO-30, BCO-90, R‑BCO‑0, R-BCO-30, and R-BCO-90 were analyzed by two-way ANOVA.

| Source of variation | SS (Type III) | DF | MS | F | P value | % total variation | Partial eta squared |
| --- | --- | --- | --- | --- | --- | --- | --- |
| Interaction | 234.600 | 2 | 117.300 | 503.3 | <0.0001 | 13.18 | 0.9883 |
| Row factor | 228.400 | 2 | 114.200 | 491.8 | <0.0001 | 12.83 | 0.9879 |
| Column factor | 1,314.000 | 1 | 1,314.000 | 5,66 | <0.0001 | 73.83 | 0.9979 |
| Residual | 2.786 | 12 | 0.2322 | — | — | — | — |

**Table S24**: Tukey’s multiple comparisons test for pairwise comparisons of the acid value among black cumin oil samples subjected to roasting and green extraction treatments.

| Tukey's multiple comparisons test | Predicted (LS) mean diff. | 95,00% CI of diff. | Adjusted P Value |
| --- | --- | --- | --- |
| BCO-0 vs. R-BCO-0 | 6,961 | 5,640 to 8,283 | <0,0001 |
| BCO-0 vs. BCO-30 | -14,04 | -15,36 to -12,72 | <0,0001 |
| BCO-0 vs. R-BCO-30 | 6,975 | 5,654 to 8,297 | <0,0001 |
| BCO-0 vs. BCO-90 | -16,17 | -17,49 to -14,84 | <0,0001 |
| BCO-0 vs. R-BCO-90 | 7,123 | 5,801 to 8,444 | <0,0001 |
| R-BCO-0 vs. BCO-30 | -21 | -22,32 to -19,68 | <0,0001 |
| R-BCO-0 vs. R-BCO-30 | 0,01383 | -1,308 to 1,335 | >0,9999 |
| R-BCO-0 vs. BCO-90 | -23,13 | -24,45 to -21,81 | <0,0001 |
| R-BCO-0 vs. R-BCO-90 | 0,1612 | -1,160 to 1,483 | 0,9981 |
| BCO-30 vs. R-BCO-30 | 21,02 | 19,70 to 22,34 | <0,0001 |
| BCO-30 vs. BCO-90 | -2,124 | -3,446 to -0,8025 | 0,0017 |
| BCO-30 vs. R-BCO-90 | 21,16 | 19,84 to 22,49 | <0,0001 |
| R-BCO-30 vs. BCO-90 | -23,14 | -24,46 to -21,82 | <0,0001 |
| R-BCO-30 vs. R-BCO-90 | 0,1473 | -1,174 to 1,469 | 0,9988 |
| BCO-90 vs. R-BCO-90 | 23,29 | 21,97 to 24,61 | <0,0001 |

**Table S25**: Two-way ANOVA of the effects of seed roasting and green extraction time on the free fatty acid content of black cumin oil. Values for BCO-0, BCO-30, BCO-90, R‑BCO‑0, R-BCO-30, and R-BCO-90 were analyzed by two-way ANOVA.

| Source of variation | SS (Type III) | DF | MS | F | P value | % total variation | Partial eta squared |
| --- | --- | --- | --- | --- | --- | --- | --- |
| Interaction | 59.250 | 2 | 29.620 | 490.4 | <0.0001 | 15.24 | 0.9879 |
| Row factor | 56.140 | 2 | 28.070 | 464.7 | <0.0001 | 14.44 | 0.9873 |
| Column factor | 272.800 | 1 | 272.800 | 4,516 | <0.0001 | 70.14 | 0.9974 |
| Residual | 0.7249 | 12 | 0.06041 | — | — | — | — |

**Table S26**: Tukey’s multiple comparisons test for pairwise comparisons of the free fatty acid content among black cumin oil samples subjected to roasting and green extraction treatments.

| Tukey's multiple comparisons test | Predicted (LS) mean diff. | 95,00% CI of diff. | Adjusted P Value |
| --- | --- | --- | --- |
| BCO-0 vs. R-BCO-0 | 2,702 | 2,028 to 3,376 | <0,0001 |
| BCO-0 vs. BCO-30 | -7,008 | -7,682 to -6,334 | <0,0001 |
| BCO-0 vs. R-BCO-30 | 2,716 | 2,042 to 3,390 | <0,0001 |
| BCO-0 vs. BCO-90 | -8,068 | -8,742 to -7,394 | <0,0001 |
| BCO-0 vs. R-BCO-90 | 2,863 | 2,189 to 3,537 | <0,0001 |
| R-BCO-0 vs. BCO-30 | -9,71 | -10,38 to -9,036 | <0,0001 |
| R-BCO-0 vs. R-BCO-30 | 0,01383 | -0,6602 to 0,6879 | >0,9999 |
| R-BCO-0 vs. BCO-90 | -10,77 | -11,44 to -10,10 | <0,0001 |
| R-BCO-0 vs. R-BCO-90 | 0,1612 | -0,5129 to 0,8352 | 0,9615 |
| BCO-30 vs. R-BCO-30 | 9,724 | 9,050 to 10,40 | <0,0001 |
| BCO-30 vs. BCO-90 | -1,06 | -1,734 to -0,3861 | 0,0021 |
| BCO-30 vs. R-BCO-90 | 9,871 | 9,197 to 10,55 | <0,0001 |
| R-BCO-30 vs. BCO-90 | -10,78 | -11,46 to -10,11 | <0,0001 |
| R-BCO-30 vs. R-BCO-90 | 0,1473 | -0,5267 to 0,8214 | 0,9735 |
| BCO-90 vs. R-BCO-90 | 10,93 | 10,26 to 11,61 | <0,0001 |

**Table S27**: Estimated shelf-life of BCO and R-BCO samples calculated from Rancimat-derived oxidative induction times using the Arrhenius equation.

|  | **BCO-0** | **BCO-30** | **BCO-90** | **R-BCO-0** | **R-BCO-30** | **R-BCO-90** |
| --- | --- | --- | --- | --- | --- | --- |
| Shelf-life (hours) | 742.56 | 1090.37 | 1323.62 | 59861.68 | 75376.00 | 111208.00 |
| Shelf-life (months) | 1.03 | 1.51 | 1.84 | 83.14 | 104.69 | 154.46 |

Folin-Ciocalteu

| c [mg gallic acid/L] | Absorbance |
| --- | --- |
| 0.2 | 0.0047 |
| 1 | 0.0244333 |
| 5 | 0.119467 |
| 10 | 0.222867 |
| 20 | 0.415233 |
| 50 | 1.01937 |
| 100 | 1.86087 |

**Fig. S1**: *Calibration curve of gallic acid for the determination of total phenolic content (TPC) via the Folin-Ciocalteu assay, with absorbance measured using a Spark photometer. Data are expressed as mean values (n = 3).*

ABTS-Assay

| c [µmol TE/kg oil] | Absorbance |
| --- | --- |
| 25 | 0.048 |
| 50 | 0.0649 |
| 90 | 0.115667 |
| 125 | 0.179967 |
| 250 | 0.342333 |
| 400 | 0.5445 |

FRAP-Assay

| c [mmol Fe(II)/kg oil] | Absorbance |
| --- | --- |
| 25 | 0.0188667 |
| 50 | 0.0333333 |
| 100 | 0.0756333 |
| 250 | 0.158767 |
| 500 | 0.292033 |
| 1000 | 0.562033 |
| 2000 | 1.1273 |

**Fig. S2**: *Calibration curves for the ABTS assay (Trolox equivalent) and the FRAP assay (ferric sulfate equivalent), with absorbance measured using a Spark photometer. Data are expressed as mean values (n = 3).*

Peroxide value

| c [µg Fe(III)/10 ml] | Absorbance |
| --- | --- |
| 5 | 0.0360667 |
| 7.5 | 0.0606667 |
| 10 | 0.100833 |
| 15 | 0.184567 |
| 20 | 0.231833 |
| 40 | 0.4634 |

**Fig. S3**: *Calibration curve for the determination of the peroxide value, with absorbance measured using a Spark photometer. Data are expressed as mean values (n = 3).*

**Fig. S4:** *Arrhenius regression plots with R^2^, Ea and predicted shelf life from OIT measurements of BCO-0, BCO-30 and BCO-90*

**Fig. S5:** *Arrhenius regression plots with R^2^, Ea and predicted shelf life from OIT measurements of R-BCO-0, R-BCO-30 and R-BCO-90*
